# High-Mobility Group Box 1-Induced Complement Activation Causes Sterile Inflammation

**DOI:** 10.3389/fimmu.2018.00705

**Published:** 2018-04-11

**Authors:** Sook Young Kim, Myoungsun Son, Sang Eun Lee, In Ho Park, Man Sup Kwak, Myeonggil Han, Hyun Sook Lee, Eun Sook Kim, Jae-Young Kim, Jong Eun Lee, Ji Eun Choi, Betty Diamond, Jeon-Soo Shin

**Affiliations:** ^1^Department of Microbiology, Yonsei University College of Medicine, Seoul, South Korea; ^2^The Center for Autoimmune Musculoskeletal and Hematopoietic Diseases, The Feinstein Institute for Medical Research, Manhasset, NY, United States; ^3^Severance Biomedical Science Institute and Institute for Immunology and Immunological Diseases, Yonsei University College of Medicine, Seoul, South Korea; ^4^Department of Anatomy, Yonsei University College of Medicine, Seoul, South Korea; ^5^Department of Pediatrics, Seoul National University Boramae Hospital, Seoul National University College of Medicine, Seoul, South Korea; ^6^Center for Nanomedicine, Institute for Basic Science (IBS), Seoul, South Korea

**Keywords:** high-mobility group box 1, complement, sterile inflammation, ischemia, hepatotoxicity

## Abstract

High-mobility group box 1 (HMGB1), a well-known danger-associated molecular pattern molecule, acts as a pro-inflammatory molecule when secreted by activated immune cells or released after necrotic cell damage. HMGB1 binds to immunogenic bacterial components and augments septic inflammation. In this study, we show how HMGB1 mediates complement activation, promoting sterile inflammation. We show that HMGB1 activates the classical pathway of complement system in an antibody-independent manner after binding to C1q. The C3a complement activation product in human plasma and C5b-9 membrane attack complexes on cell membrane surface are detected after the addition of HMGB1. In an acetaminophen (APAP)-induced hepatotoxicity model, APAP injection reduced HMGB1 levels and elevated C3 levels in C1q-deficient mouse serum samples, compared to that in wild-type (WT) mice. APAP-induced C3 consumption was inhibited by sRAGE treatment in WT mice. Moreover, in a mouse model of brain ischemia–reperfusion injury based on middle cerebral arterial occlusion, C5b-9 complexes were deposited on vessels where HMGB1 was accumulated, an effect that was suppressed upon HMGB1 neutralization. We propose that the HMGB1 released after cell necrosis and in ischemic condition can trigger the classical pathway of complement activation to exacerbate sterile inflammation.

## Introduction

High-mobility group box 1 (HMGB1) is a damage-associated molecular pattern (DAMP) molecule located in the nucleus that is secreted from activated monocytes/macrophages and released from necrotic cells (1). HMGB1 contains two DNA-binding motifs, A and B boxes, and an acidic tail. HMGB1 in the nucleus maintains chromatin structure and regulates transcription, whereas cytoplasmic HMGB1 activates inflammasome and autophagy (2). Cytoplasmic translocation and secretion of HMGB1 is regulated *via* acetylation and phosphorylation (3, 4). Extracellular HMGB1 triggers inflammation (5) and also functions as a late mediator of endotoxemia and sepsis in both animal models and human patients (6–8). Specific inhibition of endogenous HMGB1 with antagonists, such as soluble receptor for advanced end glycosylation products (sRAGE), HMGB1 A box or an anti-HMGB1 antibody (Ab), has been shown to reverse the lethality of established sepsis (9, 10). Extracellular HMGB1 alone binds to Toll-like receptor (TLR) 2, TLR4, and RAGE and activates nuclear factor (NF)-κB and extracellular signal-regulated kinase (ERK) 1/2 (11–13), thereby inducing sterile inflammation (14, 15). In addition, HMGB1 can bind to pathogen-associated molecular pattern (PAMP) molecules of lipopolysaccharide (LPS) or lipoteichoic acid (LTA) and facilitate their transfer to CD14, resulting in TLR4- or TLR2-mediated inflammation (16, 17). In sterile inflammation without bacterial substances, HMGB1 interacts with the host molecules interleukin-1β (18), chemokine (C-X-C motif) ligand 12 (CXCL12) (19), and nucleosomes (20) and augments or modifies pro-inflammatory reactions. HMGB1 acts as a pro-inflammatory cytokine mediator of sepsis; however, it induces a weak tumor necrosis factor (TNF)-α production in *in vitro* treatments (21). Therefore, the mechanism of HMGB1-mediated inflammation, as a DAMP molecule-mediated process *in vivo*, remains to be delineated.

The complement system is a first-line defense against pathogens and acts as a sensor for altered self-molecules (22, 23), triggering one of three distinct complement activation cascades: the classical, alternative, or lectin pathways (24). The classical pathway is initiated by the binding of the pattern recognition molecule C1q–IgG and IgM in immune complexes, PAMPs on microbes (25), pentraxins (PTX3) of C-reactive protein, or host apoptotic cells and debris (26). The C1 complex, formed by the binding of the proteases C1r and C1s to C1q, initially cleaves C4 into C4b and C4a and then processes C2 into C2b and C2a to form the C3 convertase. The alternative pathway is triggered directly by certain microbial cell wall components and catalyzed by factor B, whereas the lectin pathway is initiated by soluble mannose-binding lectin. The three pathways converge at the formation of C3 convertase, which catalyzes the proteolysis of C3 to execute a common terminal pathway and leads to opsonization by C3b, cell lysis *via* the formation of membrane attack complex (MAC) by C5b-9, and pro-inflammatory and anaphylactic effects mediated by C5a and C3a. The MAC inserts lytic complexes in adjacent cell membranes and mediates cellular cytotoxicity. Insertion of a complement terminal protein, C5b-9, on cell membranes over a threshold of basal complement resistance induces complement-dependent cytotoxicity to target cells or nearby bystander cells, causing lytic damage. However, subthreshold levels of the sublytic complement C5b-9 induce a variety of biological responses, including the release of pro-inflammatory mediators, the production of reactive oxygen species, the expression of adhesion molecules, and the activation of protein kinase C and ERK signaling (27). In the classical complement pathway, C1q binding to Fc region of Ig is the most common mechanism for activation. Moreover, some endogenous molecules bind to C1q in an Ab-independent fashion and activate classical complement activation, a significant process in disease progression (28).

In the present report, we demonstrate that HMGB1 binds to C1q and activates the classical complement pathway in an Ab-independent manner using molecular studies and a cell culture model system. N-acetyl-p-aminophenol (acetaminophen, APAP)-mediated hepatotoxicity in wild-type (WT) and C1q-deficient mice was used as an inflammation model to elucidate the effects of HMGB1 on C1q deposition in the liver. Immunohistochemical analysis of C1q was carried out in the presence of HMGB1 and after neutralization of HMGB1 with sRAGE or anti-HMGB1 Ab treatment. We also investigated the role of HMGB1 in activation of the classical component pathway in a mouse middle cerebral arterial occlusion (MCAO) model. We evaluated the effect of HMGB1 on the induction of complement activation *in vivo* by monitoring sublytic MAC deposition, with or without the ablation of HMGB1 by anti-HMGB1 Ab or sRAGE treatment. Collectively, our data suggest that HMGB1 is an Ab-independent C1q-binding molecule that plays an important role in classical complement activation in sterile chronic and septic inflammation.

## Materials and Methods

### DNA Constructs and Recombinant Proteins

For the recombinant HMGB1 protein, six-His-tagged recombinant human WT HMGB1, HMGB1 boxes A (aa 1-79) and B (aa 88-162), and acidic tail-deleted HMGB1 (ΔC-HMGB1, aa 1-185) were subcloned into pRSET B plasmid and produced in *Escherichia coli* BL21 (DE3) pLysE (Invitrogen) (4, 16). A six-His-tagged HMGB1 (ΔN-HMGB1, aa 11-215), a form of HMGB1 with pro-inflammatory potential (29), was subcloned into pRSET B plasmid and produced in *E. coli* BL21 (DE3) pLysE. One mM DTT was added during protein purification and preservation. Endotoxin was removed using an LPS-binding column (Thermo Fisher Scientific, Inc.) or detergent-phase separation using Triton X-114 (30). LPS concentrations were less than 0.1 EU/μg protein, as determined using the limulus amebocyte lysate assay (Sigma). In addition, HMGB1 produced in NS0 mouse myeloma cell line (Euk-HMGB1, R&D Systems) was used to confirm the study.

### Enzyme-Linked Immunosorbent Assay (ELISA) Analysis for C1q, C4b, and C5b-9 Deposition

The binding of the reduced form of HMGB1 protein, produced in *E. coli* BL20, to C1q was tested using ELISA (26). Briefly, microtiter plates (Corning) were coated with 10 µg/ml purified normal human C1q (Sigma) per well and blocked with 3% BSA-PBS. Various amounts of HMGB1 protein in 1% BSA-PBS buffer containing 0.15 mM CaCl_2_ and 0.5 mM MgCl_2_ were added to the wells and incubated for 2 h at room temperature (RT) to prevent nonspecific binding. Rabbit anti-HMGB1 Ab (1:1,500, Abcam #18256) was added for 1 h at RT after washing. HRP-conjugated anti-rabbit Ig (1:5,000, Sigma) was added to the wells for 1 h at RT. 3,3′,5,5′-tetramethylbenzidine solution (KPL) was used for color development for 15 min. Optical density values were measured at 450 nm. In the reciprocal assay, the binding of free C1q to HMGB1 protein was also tested. For this, microtiter wells were coated with HMGB1 at approximately 3–10 µg/ml, and increasing concentrations of C1q protein were added. Polyclonal rabbit anti-human C1q complement Ab (1:1,000, Dako) was used as the primary Ab. HRP-conjugated anti-rabbit Ig (1:5,000, Sigma) was used as the secondary Ab.

For the complement activation assay, we measured the complement activation products C4b and C5b-9 using ELISA after incubation of human serum with HMGB1-coated microplates. PolySorp^®^ microtiter plates (Thermo Fisher Scientific Inc.) were coated with 10 µg/ml HMGB1 (R&D Systems) or 5–10 µg/ml heat-treated (63°C for 15 min) aggregated human IgG (Sigma) as a positive control. Normal human serum (NHS) was prepared in the laboratory and preserved at −70°C (31), was diluted in gelatin veronal-buffered saline (GVB^2+^ buffer, Sigma), and added to the wells for 20 or 45 min at 37°C to measure C4b or C5b-9 deposition, respectively. After washing with a cold buffer (50 mM Tris, 150 mM NaCl, 0.1% Tween 20, pH 7.5), rabbit anti-C4b (1:1,000, Dako) or mouse anti-C5b-9 Ab (1:1,000, Quidel) was added for 1 h at 37°C. HRP-conjugated anti-rabbit Ig or anti-mouse Ig (Sigma) was used as the secondary Ab, with incubation for 1 h at RT. C1q-depleted human serum (Sigma) and purified human C1q were used as negative and positive controls, respectively.

### Binding of Complement Components to HMGB1-Coated Microspheres

Serum C1q binding to HMGB1 was tested using HMGB1-coated microspheres. Briefly, HMGB1 protein (50 µg) was conjugated with biotin using the EZ-Link Sulfo-NHS-Biotin reagent (Thermo Fisher Scientific Inc.), and non-reacted biotin was removed using a PD-10 desalting column (GE Healthcare Life Sciences). Biotinylated human IgG was used as a positive control. Streptavidin-coated microspheres (Bangs Laboratories Inc.) were incubated with biotin-labeled HMGB1 for 1 h at RT. The HMGB1- or human IgG-coated microspheres were washed three times with GVB^2+^ buffer and incubated with 10% NHS for 30 min at 37°C. After washing, the microspheres were treated with mouse anti-HMGB1 Ab (R&D Systems) and rabbit anti-C1q Ab (Dako). Alexa 594-conjugated donkey anti-mouse Ig (Invitrogen) and Alexa 488-conjugated goat-anti-rabbit Ig (Invitrogen) were used as the secondary antibodies. A confocal microscope (FV1000, Olympus) was used for observing and capturing images of the fluorescent complexes. The bindings of C3c, a degradation product of C3b after activation, and of C5b-9 to HMGB1-coated microspheres were investigated using fluorescein isothiocyanate (FITC)-conjugated rabbit anti-C3c Ab (Abcam) and mouse anti-C5b-9 Ab (Quidel). Mouse anti-HMGB1 Ab (R&D Systems) or rabbit anti-HMGB1 Ab (Abcam) was used for detecting HMGB1.

### Surface Plasmon Resonance (SPR) Assay

The analysis of HMGB1 binding to C1q was carried out using a BIAcore 2000 instrument (BIAcore Life Science), as previously described (16). A CM5 dextran sensor chip was activated with equal amounts of 0.2 M *N*-ethyl-*N′*-(3-diethylamino-propyl)-carbodiimide and 0.05 M *N*-hydroxysuccinimide. HMGB1 WT (R&D), A box or B box (produced in *E. coli* BL21) proteins (10 µg/ml) were immobilized in 10 mM sodium acetate buffer (pH 4.0) followed by 1 M ethanolamine-hydrochloride (pH 8.0) treatment to deactivate excess NHS esters. To evaluate C1q binding, C1q protein was diluted in HBS-EP buffer (10 mM HEPES, 150 mM NaCl, 3.4 mM EDTA, 0.05% Tween 20, pH 7.4) and passed over the sensor chip at a flow rate of 20 µl/min for 3 min. To regenerate the flow cell, 50 mM NaOH was passed over the chip at 30 µl/min for 10 s. An activated and blocked flow cell lacking immobilized ligand was used to evaluate nonspecific binding. For all samples, response curves were also recorded on control surfaces. Results were standardized to control values using the BIAevaluation 3.0 software (BIAcore AB).

### Complement Consumption Experiments

Complement consumption studies were carried out using the methods described in previous reports (32, 33). The consumption of human hemolytic complement was determined by the quantitative assay of residual CH_50_ in NHS after reaction with variants of HMGB1 proteins produced in *E. coli* BL21. Briefly, 50 µl of diluted NHS (CH_50_) was incubated with various amounts of HMGB1 at 37°C for 30 min. Following this, 50 µl of hemolysin-sensitized sRBCs were incubated at 37°C for 60 min followed by the addition of 30 µl of ice-cold GVB^2+^ buffer for stopping the reaction. Supernatants were harvested after centrifugation (700 × *g*, 5 min) at 4°C, and absorption values were determined at 405 nm. Spontaneous hemolysis-originating absorbance values (background) were subtracted, and the corrected absorption value of supernatant was inverted to represent complement consumption.

### Generation of MAC on MEF Cells and bEND.3 Cells

Mouse embryonic fibroblasts (MEFs) (immortalized by the 3T3 protocol, purchased from HMGBiotech) were cultured in Dulbecco’s modified Eagle medium (DMEM) supplemented with 5–10% NHS 100 U/ml penicillin, 100 µg/ml streptomycin, and 2 mM l-glutamine under 5% CO_2_ in the presence or absence of 1 µg/ml HMGB1 for 1 h at 37°C. Sublytic MAC proteins were stained using mouse anti-C5b-9 Ab and Alexa 594-conjugated donkey anti-mouse Ig for confocal microscopy. In addition, cholera toxin B-FITC (0.5 µg/ml; Sigma) was utilized to observe the cell membrane lipid raft in MEF cells.

In addition, the alteration of intercellular tight junction proteins was evaluated with an *in vitro* coculture system using the bEnd.3 mouse immortalized endothelial and the LN215 glioblastoma cell lines. bEnd.3 cells were maintained in DMEM with high glucose containing 10% FBS. bEnd.3 cells were incubated with diluted NHS in the presence or absence of 5 µg/ml reduced form of HMGB1 for 2 h. ZO-1 and occludin are transmembrane proteins that play a role in tight junction regulation (34). Tight junctions were monitored by immunofluorescence confocal microscopy with a mouse anti-ZO-1 Ab (Zymed).

### Animals

Animal procedures were carried out according to a protocol approved by the Institutional Animal Care and Use Committee (IACUC) and the Institutional Biosafety Committee of the Yonsei Laboratory Animal Research Center (YLARC, 2010-0392) and the Feinstein Institute for Medical Research (FIMR, 2013-021), Manhasset, NY, USA. Animals were allowed to acclimate for at least 2 weeks prior to initiating the experiment. All animals were housed in standard conditions (RT 22°C with a 12-h light–dark cycle) with access to regular chow and water.

### Transient MCAO Model and Tissue Immunochemistry

Seven- or eight-week-old male ICR mice (*N* = 3, Oriental Bio., South Korea) were used for the transient MCAO study, using the protocol described in a previous report (35). After 1 h of occlusion, the suture was withdrawn to restore blood flow for 4 h. To block effects of HMGB1 after MCAO operation, mice were intravenously (i.v.) administered mouse anti-HMGB1 mAb (Biolegend) (100 µg/mouse) or PBS immediately before reperfusion. Brains were removed in 1-mm thickness sectioning and fixed with 4% paraformaldehyde for 4–6 h. The tissue was fixed again with 30% sucrose in PBS for 4–5 days at RT. Samples were embedded in OCT compound (Sakura Finetek USA, Inc., Torrance, CA, USA) at −80^°^C. Tissue was sectioned at the thickness of 20 µm at −20^°^C using cryotome. The frozen sections were pre-cooled in fixative (acetone) at −20°C for 30 min and rinsed three times with PBS. Frozen sections were permeabilized with PBST and blocked for 1 h at RT with goat serum. Frozen sections were incubated overnight at 4°C with anti-C5b-9 and anti-HMGB1 Abs for confocal analysis primary antibodies and rinsed with PBS. Frozen sections were incubated with appropriate secondary antibodies conjugated with FITC, rhodamine, and Alexa Fluor^®^ 405 prepared in fluorescent Ab diluent solution (1:500, Abcam) for 2 h at RT. Frozen sections were washed with PBST and counterstained with DAPI. Images were taken using a confocal microscopy.

### APAP-Induced Hepatotoxicity Model

Nine- or ten-week-old male C57BL6/J WT (Jackson laboratory) and C1q-deficient C57BL6/J mice (FIMR, obtained from Dr. Keith Elkon, University of Washington) were treated with APAP (200–400 mg/kg of body weight) (36). Mice (9–10 weeks old) were fasted overnight and intraperitoneally (i.p.) injected with APAP solution or saline. Food was provided *ad libitum* after APAP administration. Mice were anesthetized before fasting (0 h) and 6 h after APAP administration, and blood was collected from the retro-orbital plexus; all animals were euthanized 24 h after APAP administration. Alanine aminotransferase (ALT) activity was determined using the ALT color endpoint assay (MaxDiscovery). Concentration of C3 was measured using ELISA (Alpha Diagnostic International). Soluble RAGE (sRAGE; PROSPEC, 5 µg/mouse), anti-HMGB1 Ab (provided by Kevin Tracey’s laboratory, FIMR, 5 µg/mouse), IgG (Sigma, 5 µg/mouse), or saline were i.p. injected 2 h after APAP administration. The left medial lobe of liver was fixed in 4% paraformaldehyde overnight and then 30% sucrose overnight. Paraffin-embedded liver was stained with hematoxylin and eosin (H&E) for the evaluation of necrosis and hemorrhage. For immunofluorescence assay, Tissue-Tec OCT-embedded liver was sectioned at 10-µm thickness using a microtome (Leica Biosystems), fixed with cold acetone (5 min at RT), and permeabilized using 1% Triton X-100 in 1X TBS (7 min at RT). In addition, sections were incubated for 1 h at RT in TBS + 0.1% Triton-X-100 + 10% goat serum (Invitrogen) and were stained with biotinylated mouse anti-C1q (Abcam, JL-1, 1:100) or rabbit monoclonal anti-HMGB1 (Abcam, EPR3507, 1:50) at 4°C overnight followed by the streptavidin conjugated to AF488 or goat anti-rabbit IgG conjugated with AF488 or PI. Images were obtained with LSM 510 confocal microscopy (Zeiss). Four or five mice were used for each group.

### Western Blot Analysis

To analyze the activation of C3, Western blot analysis was performed to follow the cleavage of C3 protein. NHS (diluted 1:5) was incubated with various amounts of HMGB1 for 30 min at 37°C, followed by the addition of 2× sodium dodecyl phosphate polyacrylamide gel electrophoresis (SDS-PAGE) sample buffer and then in boiling water for 5 min. The samples were resolved on 15% or 4–15% gradient gels (Biorad). After electrotransfer (wet-tank transfer system, Biorad) to nitrocellulose membranes, membranes were blocked in 5% skim milk and probed using mouse anti-human C3/C3b and anti-C3a IgG (Abcam), followed by incubation with HRP-labeled goat anti-mouse Ig (Sigma; secondary Ab) for immunoblot analysis (37). Human IgG (Sigma) was heat-treated at 65°C for 20 min to obtain aggregated Ig for use as a positive control (38). Ig-free BSA (Sigma) was used as a negative control.

To monitor *in vivo* complement activation by HMGB1, C57BL/6 mice were i.v. injected with 100 µg of HMGB1 protein. Blood samples were collected at 0, 30, and 90 min after the injection of HMGB1 in three mice, and Western blot analysis was performed to detect iC3b, a cleavage product of C3b. The relative band intensity was compared to that of the protein-specific band in control mice treated with PBS. To analyze sublytic MAC-induced ERK phosphorylation (39), we performed a Western blot analysis of phosphorylated (p)-ERK. Culture medium of MEFs was replaced with serum-free OPTI-MEM medium (Invitrogen) and incubated in 5–10% NHS in the presence of 1 or 5 µg/ml HMGB1. Cells were washed and lysed in 1X RIPA buffer containing protease and phosphatase inhibitors (Thermo Fisher Scientific) on ice for 1 h. The protein samples were analyzed using SDS-PAGE on 12% resolving gels. C5b-9, p-ERK-, and ERK-specific Abs (Cell Signaling) were used. The signals were developed with enhanced chemiluminescence (Labfrontier). To analyze serum HMGB1 and albumin from APAP liver injury model, HRP-conjugated mouse anti-HMGB1 Ab (2G7) or rabbit anti-albumin (Novus Biologicals) Ab was used. HRP signals were developed with chemiluminescence. Infrared 680-conjugated anti-rabbit Ab was detected by Odyssey (LICOR).

### Statistical Analysis

All statistical tests were performed with Graph Pad Prism 6 software (*t*-test or ANOVA). Statistical analysis of mean differences between groups was performed by unpaired two-tailed Student’s *t*-test, which was implemented in the SAS9.2 (SAS Institute Inc.). All *P*-values and *n*-values are indicated in figure legends. *P*-values of ≤0.05 were considered to be significant.

## Results

### HMGB1 Binds to C1q Protein

**Figure 1:**
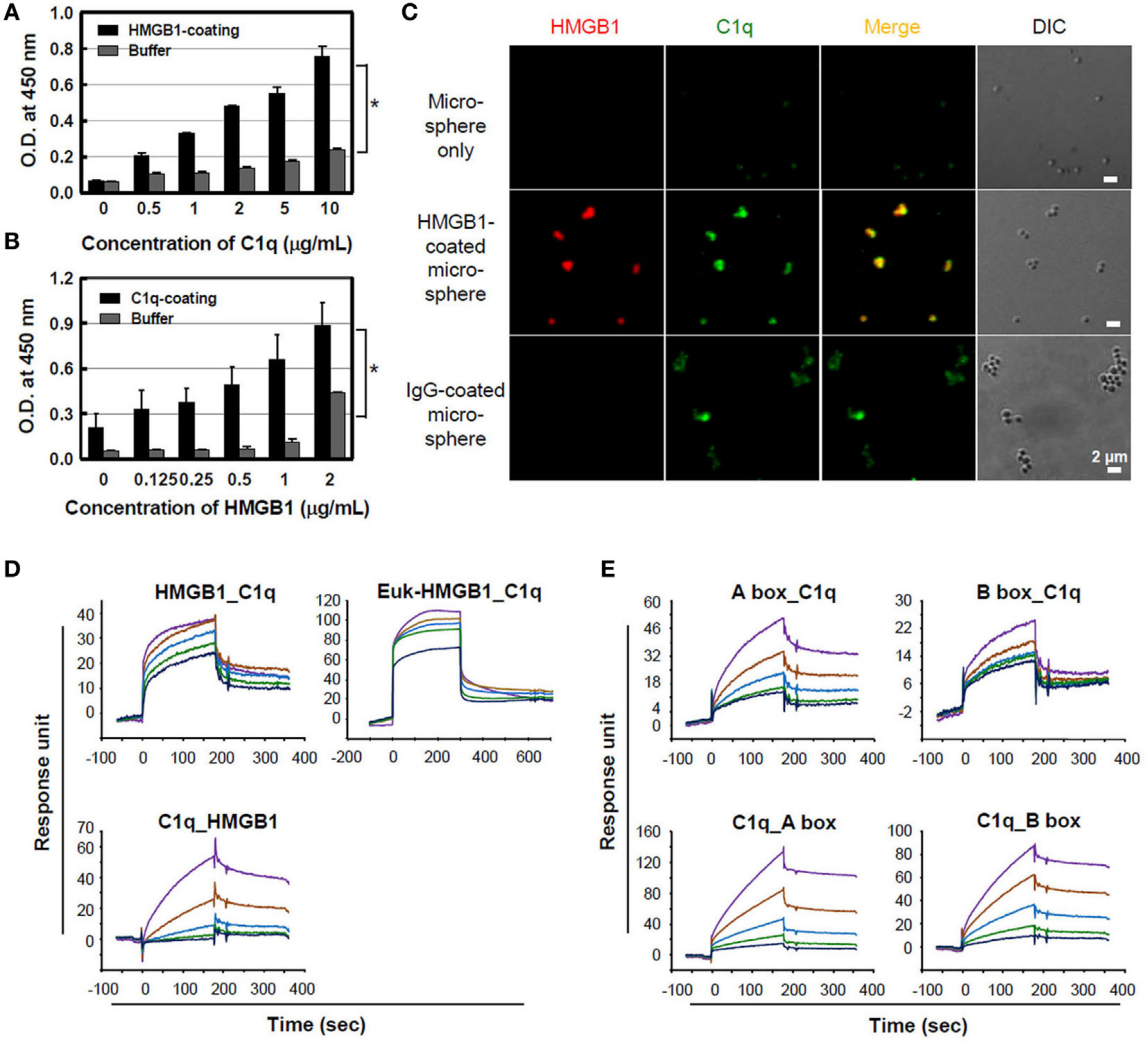
Binding of high-mobility group box 1 (HMGB1) to C1q. **(A)** Purified C1q protein (10 µg/ml) was immobilized on a microtiter plate, and different concentrations of HMGB1 protein were added for an enzyme-linked immunosorbent assay (ELISA). Buffer was used as a negative control. Data are representative of three independent experiments. Error bars are mean ± SD. *P < 0.05 by Student’s paired *t*-test. **(B)** HMGB1 (3 µg/ml) was immobilized and incubated with various concentrations of C1q for the ELISA. Data are representative of three independent experiments. Error bars are mean ± SD. *P < 0.05 by Student’s paired *t*-test. **(C)** HMGB1 protein was biotinylated and incubated with streptavidin-coated microspheres. HMGB1-coated microspheres were incubated with 10% normal human serum, which was pre-absorbed with non-coated microspheres, for 30 min at 37°C, and immunofluorescence staining was performed with anti-HMGB1 (red) and anti-C1q (green) antibodies. Biotinylated human IgG served as the positive control. Experiments were repeated at least three times and representative data are shown. Scale bar: 2 µm. **(D)** Surface plasmon resonance (SPR) analyses of HMGB1 binding to C1q protein. HMGB1 protein produced in *Escherichia coli* (HMGB1) and in eukaryotic cells (Euk-HMGB1 from R&D), left and right panels, respectively, were passed over a C1q-immobilized CM5 dextran sensor chip at concentrations of 1.8, 0.9, 0.45, 0.23, and 0.11 µM (upper panels). The reciprocal experiment of C1q binding to solid-phase HMGB1 protein at the concentrations of 200, 100, 50, 25, and 12.5 nM (lower panel) was also performed. *K_D_* = ~400 nM. **(E)** The interactions between HMGB1 A and B box proteins and C1q-immobilized chip (upper panels) and reciprocal interactions (lower panels) were also evaluated using SPR assays. Both *K_D_* = ~4 μM. Colored lines in each SPR assay represent concentrations of proteins from high to low: purple, brown, blue, green, and indigo.

Treatment with HMGB1 protein by itself has a weak pro-inflammatory activity *in vitro* (16, 21), although it is a potent effector of inflammation when released *in vivo*, suggesting that it works with other factors (40) or upregulates pro-inflammatory processes *in vivo*. The complement system is a pivotal component of the early immune response, and its activation is involved in the development of septic shock (41). For this, we hypothesized that there is a potential association between HMGB1 and complement component system since HMGB1 is a trigger molecule of inflammation, forming complex with other molecules. To determine whether HMGB1 binds to C1q, microtiter plates were coated with 10 µg/ml C1q, and HMGB1 binding to C1q was measured using ELISA. HMGB1 bound to the solid-phase C1q molecule in a concentration-dependent manner, whereas HMGB1 did not bind to the control buffer (Figure 1A). The reciprocal experiment, assessing C1q binding to solid-phase HMGB1, showed similar results (Figure 1B). No nonspecific binding was detected upon the incubation of C1q-deficient serum with HMGB1-coated wells (data not shown). Since NHS contains C1q in 113 ± 40 μg/ml (42), we tested C1q binding to HMGB1 using NHS. Biotinylated HMGB1 was cross-linked with streptavidin microspheres and incubated with 10% NHS, which was pre-absorbed with non-coated microspheres. By immunofluorescence staining, we observed that serum C1q bound HMGB1-coated microspheres with similar levels to that observed with the positive control, IgG-coated microspheres (Figure 1C).

Surface plasmon resonance analysis was performed to further evaluate the binding of HMGB1 to C1q. C1q protein was immobilized to a CM5 dextran sensor chip, and various concentrations of HMGB1 were passed over the chip. A reduced form of HMGB1 protein produced in *E. coli* exhibited binding to C1q in a concentration-dependent manner. Human HMGB1 protein produced in mouse myeloma cell line NS0 of eukaryotic cell (Euk-HMGB1) purchased from R&D Systems also bound to C1q in a dose-dependent manner. The reciprocal experiment, assessing C1q binding to solid-phase HMGB1, also indicated that C1q and HMGB1 interacted in a dose-dependent manner, confirming that HMGB1 binds to C1q (Figure 1D). Both HMGB1 A and B box proteins exhibited C1q binding on a C1q-immobilized sensor chip, and reciprocal binding studies also yielded similar results (Figure 1E). When we tested the bindings of C1q to disulfide and oxidized forms of HMGB1 in a dot ELISA assay, disulfide form of HMGB1 showed better binding to C1q than other types of HMGB1 (Figure S1A in Supplementary Material). We further evaluated whether HMGB1 and IgG bind to the same region of C1q. Results suggest that HMGB1 does not alter the interaction between IgG and C1q in ELISA (Figure S1B in Supplementary Material).

### HMGB1 Activates the Classical Complement Pathway and Terminal Complex Formation

**Figure 2:**
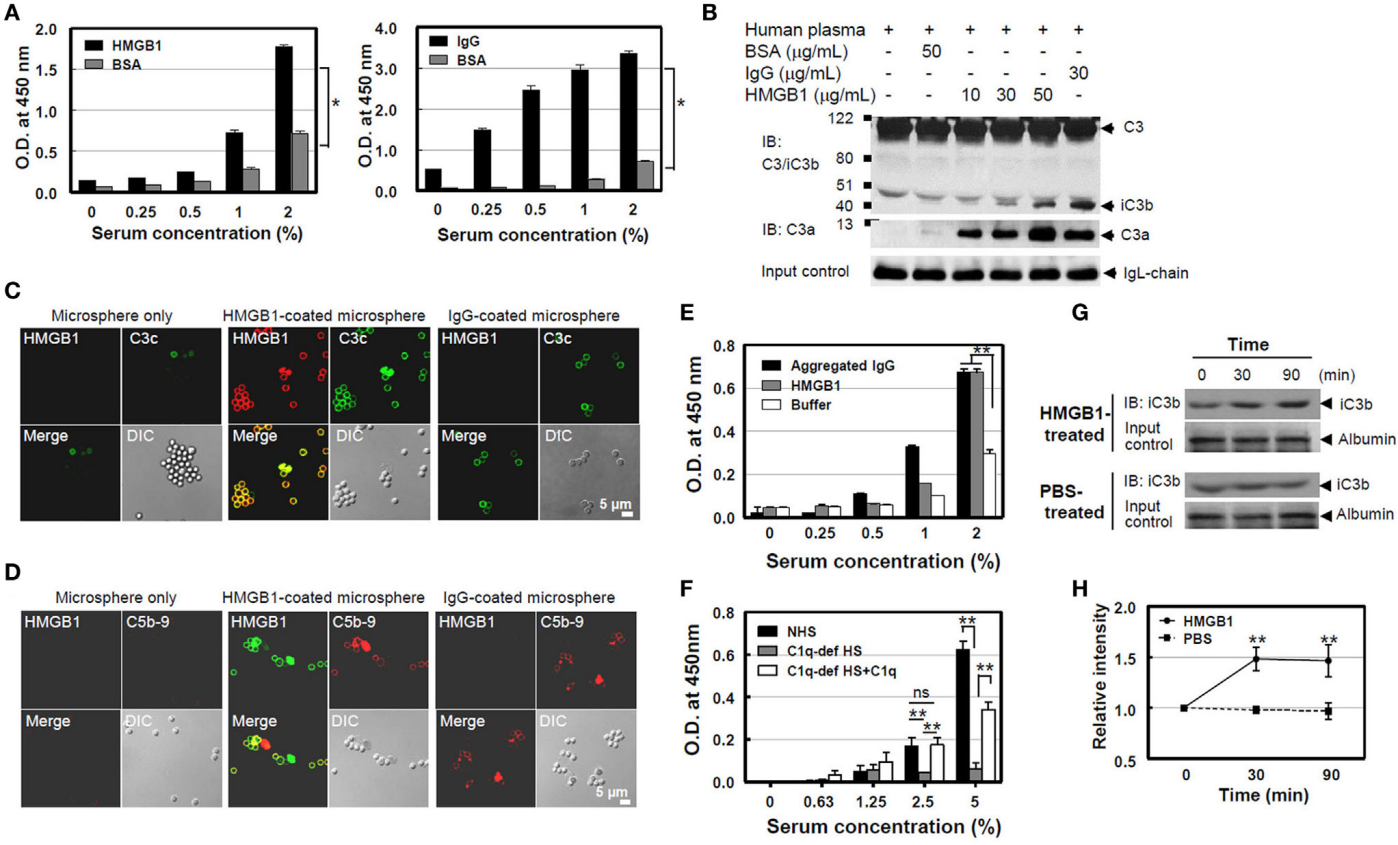
High-mobility group box 1 (HMGB1)-mediated cleavage of C4 and C3 and the formation of C5b-9 [membrane attack complex (MAC)]. **(A)** Measurement of C4b. A microtiter plate was coated with Euk-HMGB1 (10 µg/ml, R&D) and incubated with various concentrations of normal human serum (NHS) to test the deposition of C4b using an enzyme-linked immunosorbent assay (ELISA) (left). Human IgG (10 µg/ml) was used as the positive control (right). **(B)** To determine C3a and iC3b, the cleavage product of C3b. Fifty microliters of NHS (diluted 1:5) was incubated with increasing amounts of HMGB1 for 30 min at 37°C, then performed Western blot analysis using anti-C3/iC3b or anti-C3a antibodies. Aggregated human IgG and Ig-free BSA served as the positive and negative controls, respectively. Ig light chain (IgL) was used as input control. **(C,D)** Measurements of complement activation products of C3c, the cleaved form of C3b, and C5b-9. Biotinylated HMGB1s were incubated with streptavidin-coated microspheres in 10% NHS, which was pre-absorbed with non-coated microspheres, for 30 min at 37°C. Immunofluorescence staining was performed with anti-HMGB1 and fluorescein isothiocyanate-conjugated anti-C3c antibody (Ab). For C5b-9 deposition, immunofluorescence staining was performed with anti-HMGB1 (green) and anti-C5b-9 Ab (red). Biotinylated human IgG was used as the positive control. **(E)** A microtiter plate was coated with HMGB1 (10 µg/ml) and incubated with various concentrations of NHS to test the deposition of C5b-9 using an ELISA. Aggregated human IgG was used as the positive control. **(F)** C1q-dependent MAC formation. A microtiter plate was coated with HMGB1 (10 µg/ml) and incubated with various concentrations of NHS or C1q-depleted human serum (C1q-dep HS) to test the deposition of MAC formation using an ELISA for C5b-9. To restore the effect of C1q, 20 µg/ml of C1q was added to C1q-depleted human serum. ns: not significant. **(G,H)** Measurement of complement activation after HMGB1 injection in mice. C57BL/6 mice were intravenously injected with 100 µg of HMGB1 or PBS to observe if HMGB1 can activate complement *in vivo* (*N* = 3 per group). Blood samples were collected at 0, 30, and 90 min after the injection. iC3b (arrow) was detected using Western blot analysis of serum sample from one representative mouse. Relative band intensity of iC3b was analyzed. All data shown here are representative of at least three independent experiments with similar results. Error bars are mean ± SD. *P < 0.05, **P < 0.01 by Student’s paired *t*-test **(A)** or two-way ANOVA **(E,F,H)**.

It is known that serum complement proteins play an important role in resistance to infection, promoting the formation of a pore-forming MAC on the bacterial cell wall that causes lytic damage. Since C1q is an initiator for complement cascade with C1 and C1 activation induces C4 cleavage to C4b, further promoting complement activation, we measured C4b product formation using ELISA after the addition of NHS on HMGB1-coated plates to evaluate C4 activation by HMGB1. C4b deposition increased in an NHS concentration-dependent manner, similar to that seen in the IgG-coated plates (positive control; Figure 2A). We next tested C3 cleavage using Western blot analysis after the incubation of HMGB1 with NHS. C3 in NHS was cleaved into C3a and iC3b, one of cleaved products of C3b, and the cleaved-product formation was proportional to the HMGB1 concentration (Figure 2B). We also measured levels of another complement activation product, C3c, the cleaved form of C3b, using microsphere beads. HMGB1-coated microspheres were incubated with NHS, and the downstream activation product of C3c was detected using an anti-C3c Ab conjugated with fluorescein isothiocyanate (FITC). The levels of the cleaved C3c product were similar to that in the positive control of IgG-coated microspheres (Figure 2C). In addition, we could observe the formation of later step products of C5b-9 neoantigen on the surface of microspheres using confocal microscopy (Figure 2D). We, next, tested the formation of C5b-9 neoantigen using ELISA. Increasing concentrations of NHS were added to HMGB1-coated microwell plates for 45 min, followed by the addition of an anti-C5b-9 Ab to monitor the accumulation of C5b-9 on the plates after washing. The binding of C5b-9 to the microwells was dependent on the dose of NHS (Figure 2E). C5b-9 neoantigen formation was profoundly decreased in C1q-deficient human serum to HMGB1-coated microwells as expected, but it was significantly restored when added with 20 µg/ml of C1q protein (Figure 2F).

Moreover, we tested whether HMGB1 induces the complement activation in mouse. *In vivo* measurement of complement activation by HMGB1 was performed after injection of HMGB1 into a mouse model. Three C57BL/6 mice were i.v. injected with 100 µg of HMGB1 protein, and blood samples were collected at 0, 30, and 90 min after injection. iC3b, a cleavage product of C3b, could be detected at 30 min. The relative band intensity of iC3b in mice treated with HMGB1 was increased approximately 1.5-fold (1.505 and 1.499, respectively) at 30 and 90 min, compared to that in a control mouse treated with PBS (Figures 2G,H). These data suggest that WT HMGB1 protein binds to C1q and activates C1q-dependent classical complement pathway, leading to the terminal activation of C5b-9.

### HMGB1 B Box Is Main Domain to Induce the Lytic Activity of Complement

**Figure 3:**
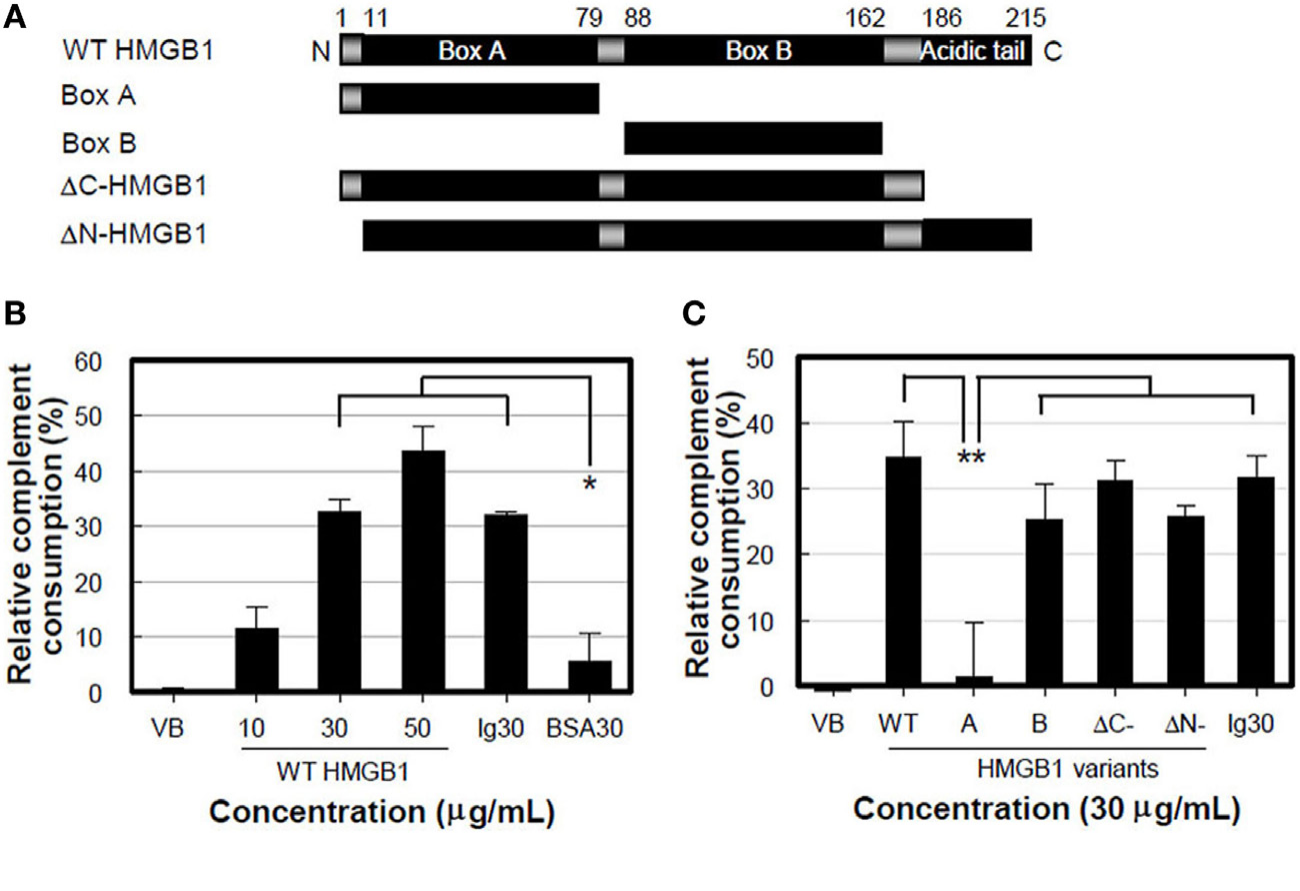
High-mobility group box 1 (HMGB1) B box activates complement cascade. **(A)** Schematic overview of recombinant HMGB1 proteins used: wild-type (WT) HMGB1, boxes A (aa 1-79) and B (aa 88-162), ΔC-HMGB1 (aa 1-185), and ΔN-HMGB1 (aa 11-215). **(B,C)** Complement consumption assessment. WT HMGB1 proteins **(B)** or HMGB1 variants **(C)** were incubated with the diluted normal human serum (NHS) containing CH_50_ activity in GVB^2+^ buffer for 30 min at 37°C. After complement consumption by HMGB1, sRBCs were added in the consumed NHS for 30 min at 37°C. Aggregated human IgG and IgG-free BSA (each 30 µg/ml) were used as the positive and negative controls, respectively. Data shown are the mean ± SD of three independent repeats. *P < 0.01 vs. BSA30, **P < 0.001 by two-way ANOVA with Bonferroni correction.

We performed a complement consumption assay to further test our hypothesis that complement hemolytic activation can be mediated by HMGB1 using various truncated forms of HMGB1 (Figure 3A). WT HMGB1 was pre-incubated with CH_50_ NHS and then hemolysin-sensitized erythrocytes (EAs) were added to measure the residual complement lytic activity. The complement consumption induced by WT HMGB1 was increased in a dose-dependent manner, compared to that induced by the negative control of BSA (Figure 3B). The levels of complement consumption were 36.8 and 43.4% in the presence of 30 and 50 µg/ml HMGB1, respectively. This suggests that HMGB1 activates complement hemolytic activity.

We further determined the domain(s) of HMGB1 important for activating the complement system. HMGB1 A box protein failed to induce complement consumption (Figure 3C), although it bound to C1q efficiently (Figure 1E). ΔC-HMGB1, the form of HMGB1 lacking the acidic tail, induced activity similar to that of WT HMGB1 in complement consumption, indicating that the acidic tail of HMGB1 is not critical for complement activation. HMGB1 has a cleavage site between amino acids 10 and 11, where vascular endothelial cell-associated thrombomodulin–thrombin complexes bind. This cleavage results in the formation of ΔN-HMGB1 (amino acids 11-215), a less potent form of HMGB1 that participates in HMGB1-induced coagulation (29). ΔN-HMGB1 showed significant complement consumption, indicating that the 10 N-terminal residues are not involved in complement activation (Figure 3C). Nonetheless, B box protein itself induced significant complement consumption. These data suggest that the B box domain of HMGB1 is critical for the activation of complement.

### HMGB1-Mediated MAC Formation and Its Effect to Cell Signaling

**Figure 4:**
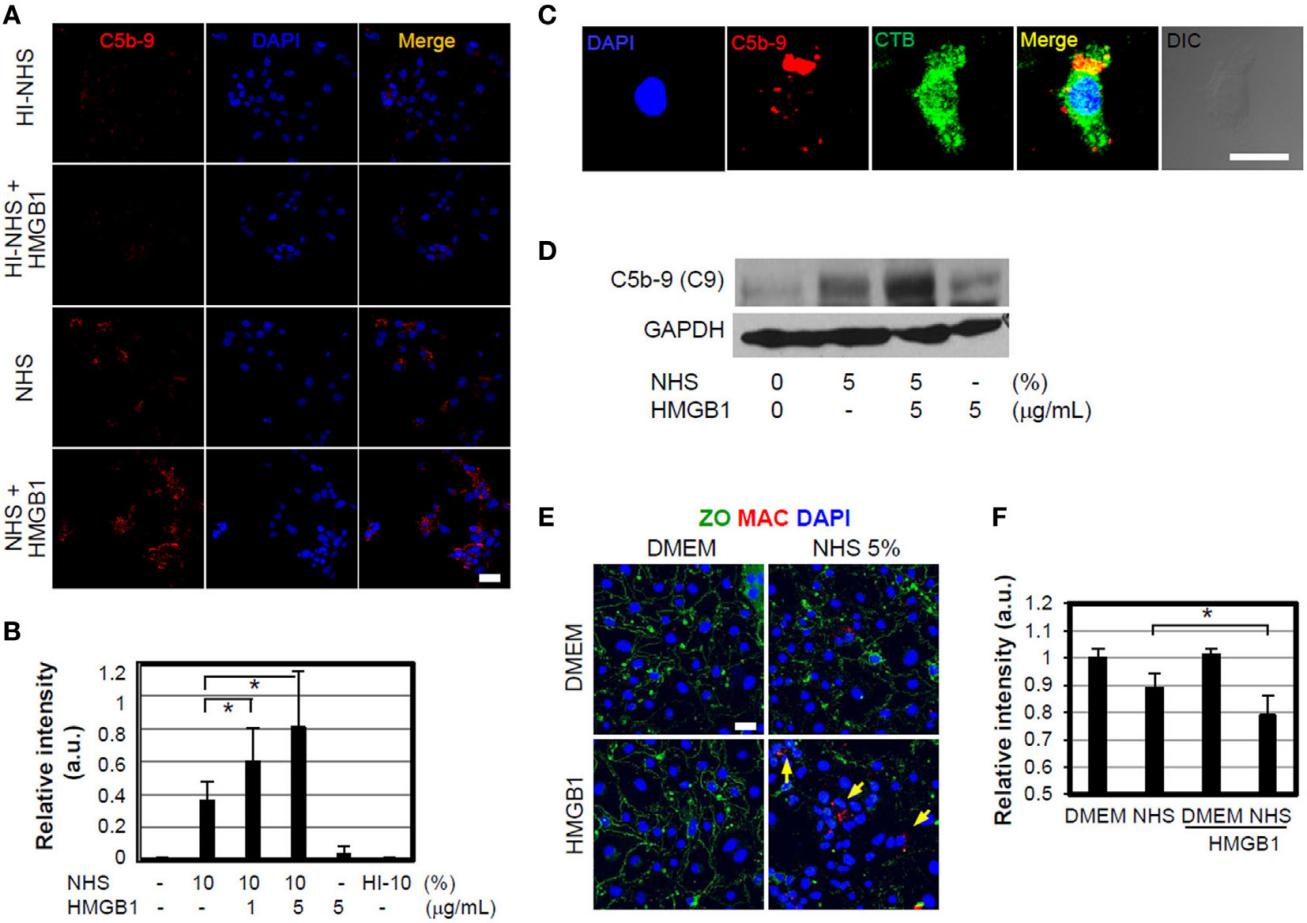
High-mobility group box 1 (HMGB1)-mediated membrane attack complex (MAC) formation and its effect to cell signaling. **(A,B)** MEFs were cultured in DMEM containing 10% normal human serum (NHS) in the presence or absence of 1 µg/ml HMGB1 for 1 h at 37°C. Sublytic MAC proteins were stained using anti-C5b-9 antibody (Ab) (red) and observed by confocal microscopy. Blue: DAPI. Heat-inactivated (HI) NHS was used. Scale bar, 10 µm **(A)**. MEFs were incubated with 10% NHS in the presence of different concentrations of HMGB1, and then the mean relative intensity of fluorescence of 10 visual fields was calculated **(B)**. Error bars are mean ± SD. *P < 0.05 by Student’s paired *t*-test. **(C)** MEFs were cultured in DMEM containing 5% NHS in the presence of 1 µg/ml HMGB1 for 1 h at 37°C. To observe MAC formation, MEFs were fixed and mouse anti-C5b-9 Ab was used for immunofluorescent analysis. Cholera toxin B-FITC (CTB 0.5 µg/ml, Sigma) was utilized to observe the cell membrane lipid raft using confocal microscopy. Scale bar, 10 µm. **(D)** bEnd.3 cells were cultured in the presence of 5% NHS and/or 5 µg/ml HMGB1 and MAC formation was observed using Western blot analysis. **(E,F)** MAC formation. bEND.3 cells and LN215 cells were cocultured and incubated with DMEM containing 5% NHS in the presence or absence of 5 µg/ml HMGB1 for 16 h, and the alteration of tight junction (green line) and sublytic MAC deposition (red fluorescence, arrow) was observed by using confocal microscopy. Anti-ZO-1 Ab and anti-C5b-9 Abs were used for the study. Scale bar = 10 μm **(E)**. The mean relative intensity of fluorescence of six visual fields of zonal occluding (ZO) was calculated **(F)**. Error bars are mean ± SD. *P < 0.05 by Student’s paired *t*-test.

We also investigated whether HMGB1 could induce MAC or sublytic MAC insertion on cell membranes of nearby bystander cells after complement action. MEFs were incubated with 1 or 5 µg/ml HMGB1 and 10% NHS for 1 h at 37°C and immunostained to detect MAC deposition. MAC was detectable on the cell surface (Figures 4A,B) and some inside the cell due to endocytosis or vesiculation (Figure 4C) (43), and its deposition was increased at the higher concentration of HMGB1. However, heat-inactivated serum did not induce MAC formation occurred by HMGB1 as expected. HMGB1 also induced the sublytic MAC deposition on bEND.3 cells, an immortalized mouse endothelial cell line, when these cells were cultured in the presence of HMGB1 (Figure 4D), demonstrating complement activation by HMGB1.

We next used a fluorometric assay to assess cell membrane integrity, quantitatively triggered by sublytic MAC, using an *in vitro* model of the blood–brain barrier (BBB) in which bEnd.3 cells are cocultured with LN215 astrocytoma cells (44). Tight junctions were monitored by immunofluorescence confocal microscopy using a mouse anti-zonal occludin (ZO)-1 Ab. The addition of HMGB1 to 5% NHS significantly disrupted ZO-containing tight junctions and accumulation of MAC (Figures 4E,F). We evaluated the effect of HMGB1-mediated MAC deposition on the cell surface on membrane integrity. bEnd.3 cells were pre-stained with 3 µM biscarboxyethyl-5(6)-carboxyfluorescein (BCECF) and cultured in media containing various concentrations of NHS, in the presence or absence of 5 µg/ml HMGB1, and the BCECF fluorescence in the supernatants was then measured as an indicator of membrane leakage (44). The release of BCECF into the supernatant was increased by the addition of HMGB1 to 20% NHS (Figure S1C in Supplementary Material). These results suggest that HMGB1 may cause vascular permeability and BBB disruption in brain ischemia by promoting complement activation, consistent with our data and those described in a previous study (45). We next used two animal models of MCAO ischemia and reperfusion model and APAP-induced hepatotoxicity model, which represent oxidative stress model and direct hepatotoxicity model, respectively, to support *in vivo* experiments of HMGB1-mediated complement activation.

### MAC Deposition Is Reduced by Anti-HMGB1 Neutralizing Ab in MCAO Ischemia Mouse Model

**Figure 5:**
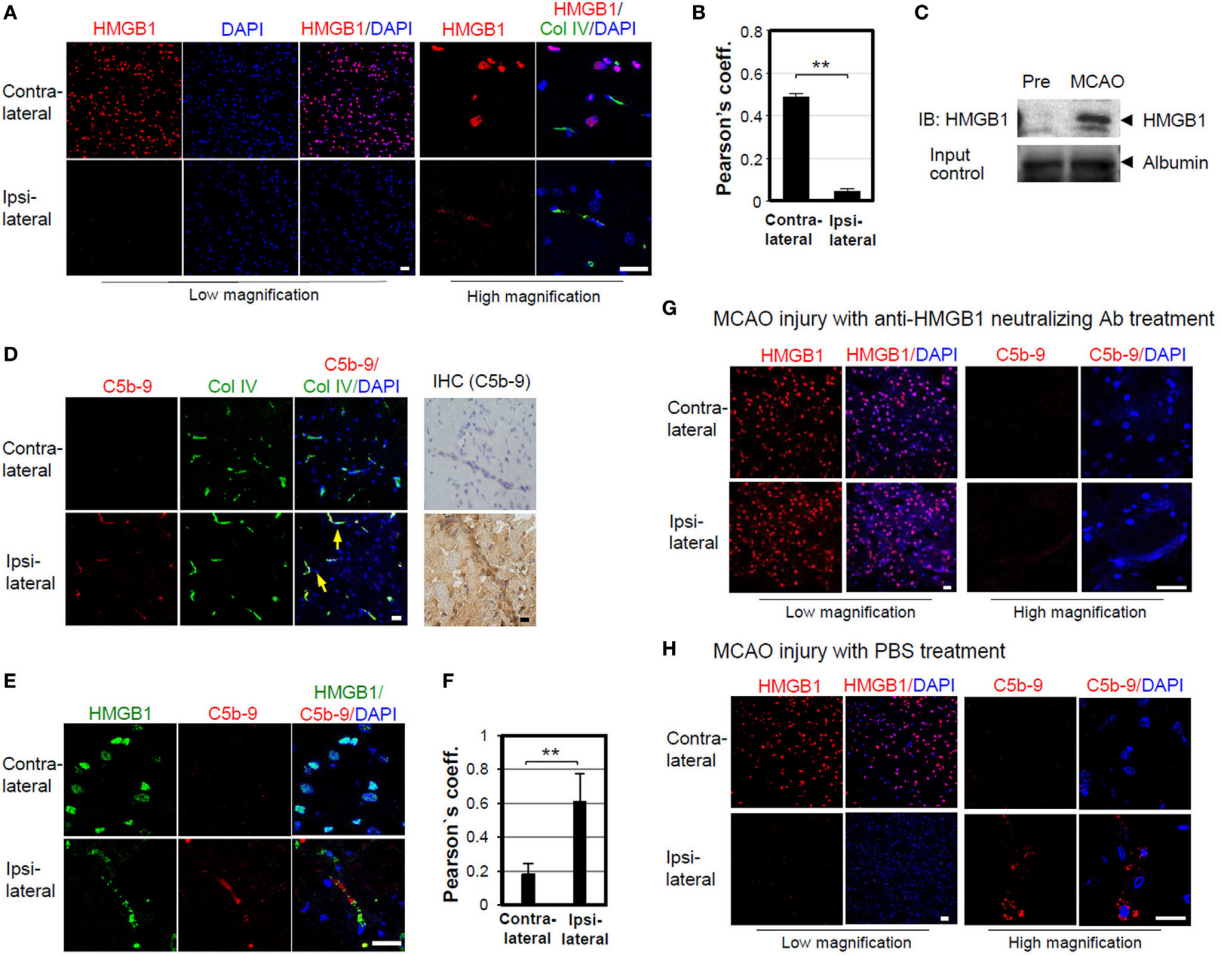
Membrane attack complex (MAC) deposition is reduced by high-mobility group box 1 (HMGB1) neutralizing antibody (Ab) in middle cerebral arterial occlusion (MCAO) ischemia mouse model. **(A)** HMGB1 staining of ICR mouse brain 4 h after MCAO. Contralateral and ipsilateral brain sections were stained for HMGB1 and collagen (Col) IV, which visualizes most blood vessels and capillaries. The images captured at low and high magnifications are shown. **(B)** Pearson’s coefficient for the overlapping of HMGB1 and DAPI in low magnification of three fields was calculated. Error bars are mean ± SD. **P < 0.01 by Student’s paired *t*-test. **(C)** Serum HMGB1 was detected using Western blot analysis after MCAO. **(D)** Sublytic MAC deposition (arrow) was observed in mouse brain, 4 h after MCAO. Brain sections were immunostained against C5b-9 for confocal microscopy and immunohistochemistry. **(E)** Brain sections were stained against HMGB1 and sublytic MAC after MCAO, and their colocalization could be detected in the ipsilateral brain region. **(F)** Pearson’s coefficient for overlapping of HMGB1 and sublytic MAC of 42 random visual fields was calculated. Error bars are mean ± SD. **P < 0.01 by Student’s paired *t*-test. **(G,H)** Mice were intravenously injected with anti-HMGB1 neutralizing Ab (100 μg/mouse) or PBS 4 h after MCAO reperfusion injury. Mouse brain sections were immunostained for HMGB1 (low magnification) and MAC deposition for detail observation (high magnification). All scale bars, 20 µm. Four mice were used for the study.

High-mobility group box 1 is an important mediator of BBB damage in ischemia-induced disruption (45). We tested the role of HMGB1 in BBB disruption by complement activation in mouse brain ischemia and reperfusion experiments, by inducing MCAO injury and then monitoring the release of HMGB1 from brain parenchymal cells. Intracellular HMGB1 was significantly reduced in the ipsilateral side of brain, 4 h after MCAO injury, whereas the contralateral side was 10.5-fold positive for HMGB1 staining (Figures 5A,B). Consistent with these data, the band intensity of HMGB1 level in serum was increased by 5.6-fold, 4 h after MCAO (Figure 5C). Next, we tested the formation of MAC complexes. MAC complexes were detected in brain vascular areas stained with a type IV-collagen Ab in the ipsilateral side after MCAO injury (Figure 5D). MAC deposition and HMGB1 colocalization in ipsilateral brain were increased by 2.3-fold (Figures 5E,F). These data suggest that ischemia-mediated HMGB1 accumulation in brain vascular cells induces complement activation and C5b-9 accumulation. To evaluate the effect of HMGB1 neutralization on the deposition of MAC, we injected mice with an anti-HMGB1 neutralizing Ab after induction of MCAO. Intact nuclear HMGB1 staining in ipsilateral brain, similar to that in the contralateral section, was detected in mice with MCAO injury; moreover, MAC deposition appeared sparse (Figure 5G), compared to MCAO injury with PBS treatment group (Figure 5H).

### HMGB1-Induced Complement Activation Is Decreased in C1q-Deficient Mice of APAP-Induced Hepatotoxicity Model

**Figure 6:**
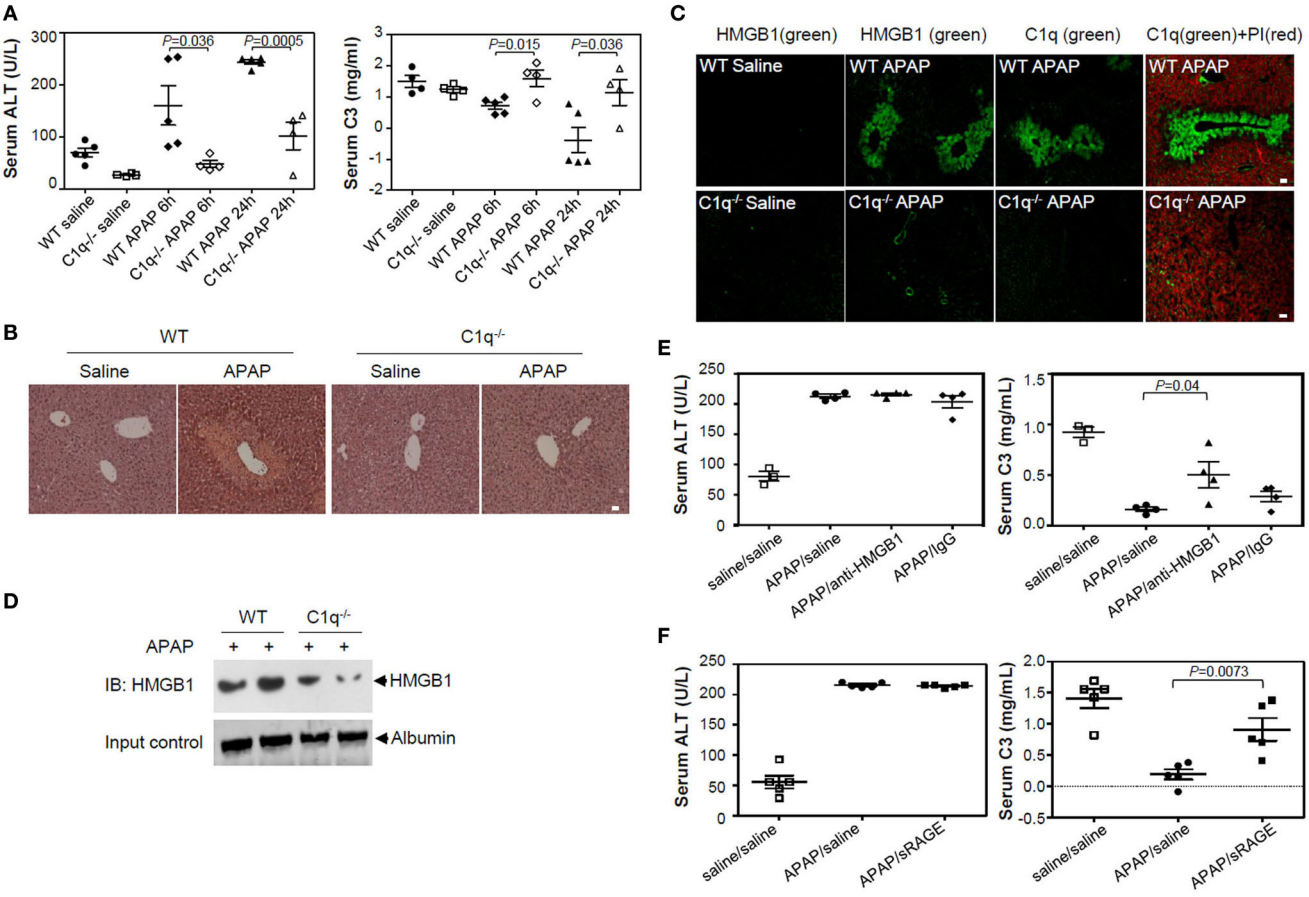
High-mobility group box 1 (HMGB1)-induced complement activation is decreased in C1q-deficient mice of APAP-induced hepatotoxicity model. **(A)** Overnight fasted wild-type (WT) or C1q^−/−^ mice (9–10 weeks old, male) were intraperitoneally (i.p.) treated with 400 mg/kg of APAP or saline. After 0, 6, and 24 h, mouse serum samples were collected. Serum alanine aminotransferase (ALT) activity (left) and C3 levels (right) were determined using ALT color endpoint assay and enzyme-linked immunosorbent assay, respectively. Each dot represents each mouse with triplicates per sample (four or five mice per group). **(B,C)** Mice were euthanized 24 h after APAP administration, and the left medial lobe of liver was fixed in 4% paraformaldehyde and 30% sucrose. Liver tissue was stained with hematoxylin and eosin for evaluation of necrosis and hemorrhage **(B)**. Tissue-Tec OCT-embedded liver was stained with anti-C1q and anti-HMGB1 antibodies (Abs) for immunofluorescence analysis **(C)**. **(D)** Serum HMGB1 protein from WT and C1q^−/−^ mice was detected using Western blot analysis 24 h after APAP injection. **(E)** Anti-HMGB1 (2G7) or mouse IgG (5 μg/mouse) was i.p. administrated immediately after APAP administration to block HMGB1. After 24 h, mouse serum samples were collected and ALT activity (left panel) and concentration of C3 (right panel) were determined. Saline was used as a negative control. **(F)** HMGB1 was i.p. injected with sRAGE (5 µg/mouse) as described in **(E)**. *N* = 4–5 mice/group. Data = mean ± SEM. Student’s *t*-test (unpaired two-tailed) was used to calculate the *P*-value. All scale bars, 20 µm. Data are representative of three **(A–E)** or two **(F)** independent experiments.

**Figure 7:**
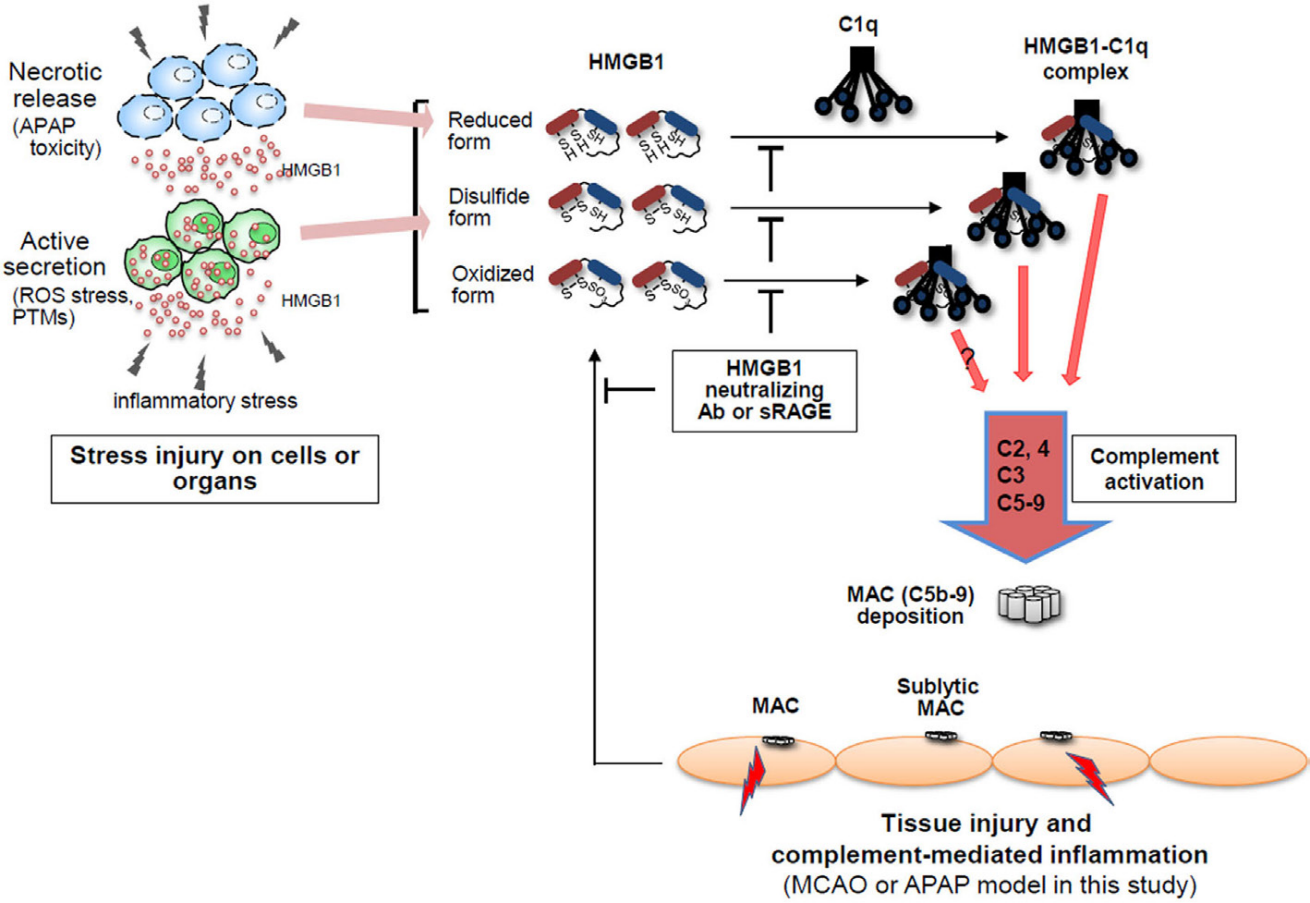
High-mobility group box 1 (HMGB1)-induced complement activation model. HMGB1 protein is actively secreted or passively released by stress injury on cells or organs in the forms of three different redox statuses, which may differently modulate immunological activities. Extracellular HMGB1 can activate the classical pathway of complement system in an antibody (Ab)-independent manner after binding to C1q, resulting in forming C5b-9 membrane attack complexes (MAC) where HMGB1 is accumulated. Thus, HMGB1-induced complement activation is proposed to be able to exacerbate sterile inflammation.

We investigated the effect of HMGB1 on complement activation *in vivo* using an APAP-induced liver injury model in C1q-deficient mice. WT and C1q-deficient mice were injected with APAP (400 mg/kg of body weight) to induce liver damage and promote extracellular release of HMGB1. Levels of ALT and C3 in sera were measured 0, 6, and 24 h of APAP treatment to monitor liver cell damage and complement activation, respectively. Serum ALT levels were significantly elevated in a time-dependent manner, especially 6 and 24 h after APAP treatment in WT mice (Figure 6A), and liver cells were damaged mainly at the centrilobular region, as indicated by the data from H&E staining of liver sections (Figure 6B, left panel). By contrast, C1q-deficient mice had significantly lower ALT levels than that of WT mice after APAP treatment, and analysis of the liver sections indicated smaller areas of necrosis (Figure 6A left and Figure 6B right). C3 levels decreased in WT mice over time after APAP treatment, while C3 persisted at higher levels in C1q-deficient mice (Figure 6A, right panel), indicating impaired consumption of serum C3 after APAP treatment in C1q-deficient mice. HMGB1 was detected in necrotic areas of the centrilobular region of the livers in WT mice, where C1q was deposited (Figure 6C upper). However, HMGB1 was less readily detected (faint staining in a smaller area of the centrilobular regions) in livers from C1q-deficient mice (Figure 6C, lower panel). Moreover, the serum HMGB1 level was lower in C1q-deficient mice, compared to that in WT mice (Figure 6D). We tested whether blocking HMGB1 activity could restore serum C3 levels in WT mice after APAP treatment. An HMGB1 neutralizing Ab and antagonist, sRAGE, were injected into WT mice immediately following APAP injection. Neutralizing Ab treatment resulted in the significant recovery of C3 levels, 24 h after Abs-mediated HMGB1 blockage, compared to that in the control IgG-treated mice (Figure 6E). We also found similar results in WT mice treated with sRAGE (Figure 6F). Even though the blockade of HMGB1 did not change the level of ALT 24 h after APAP treatment (Figures 6E,F, left), serum C3 level in WT mice after APAP treatment was restored (Figures 6E,F, right). These results confirm the role of HMGB1-induced complement activation in the APAP liver cell damage model, occasionally in the C1q-dependent manner. In summary, these data show that HMGB1 could activate Ab-independent and C1q-mediated classical complement pathway. Targeted therapy against HMGB1 may ameliorate the sterile inflammation promoted by HMGB1-mediated complement activation (Figure 7).

## Discussion

High-mobility group box 1 is a well-known mediator of sepsis. HMGB1 augments the inflammatory response by binding to bacterial substances such as LPS and LTA and stimulates TNF-α production (16, 17). HMGB1 has been reported to induce weak TNF-α production *in vitro*, but the initial inflammatory mechanism through which HMGB1 mediates sterile inflammation without bacterial factors remains to be elucidated. In this study, we show that HMGB1 binds to C1q molecules and subsequently activates C4 and C3, ultimately inducing MACs, by activating the classical complement pathway. Conventionally, the classical complement pathway is activated by C1q binding to Ag–Ab complexes. C1q can also be activated by an Ab-independent classical pathway, through the PTX3 family of serum plasma proteins, including C-reactive protein, serum amyloid protein, PTX3, and other membrane proteins like CD91 (46). Based on the data, we propose that HMGB1 is another ligand capable of Ab-independent classical pathway activation, and extracellular release of HMGB1 by oxidative stress or ischemic damage could induce sterile inflammation by binding to C1q molecule.

High-mobility group box 1, however, is a redox-sensitive protein that contains three conserved cysteine residues: Cys^23^, Cys^45^, and Cys^106^ and shows different functions depending on the oxidation status of three cysteines (47). Reduced form of HMGB1 (all thiol HMGB1) has all three cysteine residues in the thiol state, disulfide form of HMGB1 has an intramolecular disulfide bond between Cys^23^ and Cys^45^ with Cys^106^ in the thiol state, and oxidized form of HMGB1 has all three cysteines in the hyperoxidized sulfonic acid state. All three kinds of HMGB1 show the binding to C1q (48). We used “reduced form of HMGB1” *in vitro* study. We observed that “disulfide form of HMGB1” also showed the complement activation (data not shown), and further investigation is necessary for the complement activation by oxidized form of HMGB1.

In an APAP-induced acute liver injury model, the metabolic product of APAP, *N*-acetyl-*p*-benzoquinoneimine, induces mitochondrial dysfunction and eventual cell necrosis (49, 50), as well as the release of DAMP molecules like HMGB1 and heat-shock proteins (51). HMGB1 released from damaged hepatocytes promotes inflammatory processes, and the neutralization of HMGB1 can attenuate liver injury (52). In our study, APAP induced cellular necrosis in the centrilobular regions, where HMGB1 and C1q molecules were deposited, as well as high levels of serum HMGB1 in WT mice. Moreover, serum C3 was significantly reduced, but could be restored when HMGB1 was blocked by sRAGE treatment and neutralizing anti-HMGB1 Ab, suggesting that C3 consumption is HMGB1-mediated. By contrast, C1q-deficient mice showed attenuated centrilobular necrosis and HMGB1 staining after APAP treatment and higher levels of serum C3. A previous report showed that APAP injection elevates serum HMGB1 levels (53). Singhal et al. (36) showed that complement is activated after APAP-induced liver injury, and C3 fragments are deposited at centrilobular regions. Our findings, along with these data, suggest that HMGB1 is the missing link between complement activation and liver injury in the APAP-induced liver injury mouse model.

We further confirmed the role of HMGB1 in complement activation using another sterile inflammation mouse model, MCAO. HMGB1 is severely depleted in neurons of infarction cores, consistent with a previous report (54), and some HMGB1 was deposited in the microvascular wall of the ipsilateral side, 4 h after MCAO injury. Complement activation products of C5b-9 were significantly colocalized with the sites of HMGB1 deposition. However, HMGB1 was not depleted in neurons, and the deposition of C5b-9 was not readily detectable in the microvascular wall when HMGB1 was neutralized after MCAO injury. BBB is disrupted to cause brain edema during the early phase of ischemic brain injury. Our findings, along with the observation that anti-HMGB1 Ab treatment protects BBB from ischemia-induced disruption in rats (45), lead us to conclude that HMGB1-mediated complement activation plays an important role in the permeability of BBB. Recently, C5a plays an important role in regulating HMGB1 release in sepsis (55). C5a alters BBB integrity in a human *in vitro* model of systemic lupus erythematosus and experimental murine lupus (56, 57). Neutralization of C5a prevents breakdown of the BBB in experimental sepsis and suppression of HMGB1 release (58, 59), suggesting that HMGB1-mediated complement activation is an important therapeutic target.

Wild type, ΔC-HMGB1, ΔN-HMGB1, and the B box could induce complement consumption. It is interesting that HMGB1 B box, but not A box, could activate the classical complement pathway, even though both A and B boxes could bind to C1q. Amyloid-β (Aβ) activates the pathway in an Ab-independent manner (28), and aggregated Aβ shows multimeric binding to C1q to activate the classical pathway (60). The pro-inflammatory effect of the B box domain may be due to the tendency of B box to dimerize or oligomerize more readily than A box (61) and other data from our laboratory (unpublished observations), suggesting that multimeric binding of B box to C1q occurs in initial complement activation. The interaction study of HMGB1 B box with C1q will help to clarify the molecular basis of HMGB1-C1q binding. In C1q- and IgG-binding assay, HMGB1 did not inhibit their association, suggesting that HMGB1 does not displace IgG from C1q. It is possible that the six globular heads of C1q serve as multiple binding sites for HMGB1 and IgG individually.

Membrane attack complexes are important in mediating complement-related cell lysis or tissue damage by extensive deposition (39), whereas a suboptimal-dose deposition of MACs can contribute to sublytic effects of inflammatory responses. These sublytic effects are mediated *via* the activation of several signaling events, including NF-κB, protein kinase C, and MAPK pathway activation, Ca^++^ influx/mobilization (27, 39). In our study, co-incubation of HMGB1 with NHS resulted in either significant cell lysis or sublytic effects (ERK phosphorylation) (data not shown). HMGB1 is one of the main factors contributing to ischemia–reperfusion injury and is a potential therapeutic target for ischemia (45, 62). Our data show that extracellular HMGB1 generated during ischemic injury may activate the classical complement pathway and induce lytic or sublytic MAC deposition, which creates a subclinical condition (39, 63). Considering that HMGB1, 2, and 3 are very homologous, and further investigation is necessary to determine whether HMGB2 and 3 have similar functions in the classical pathway of complement activation.

The binding of HMGB1 and C1q, however, reciprocally regulates human macrophage polarization (48). Son et al. show that HMGB1 interacts with C1q (*K_D_* = 200 nM), which tail binds to leukocyte-associated Ig-like receptor (LAIR)-1 resulting in SHP-1 recruitment for anti-inflammatory response (48). These contradictory observations show that HMGB1 could show both pro- and anti-inflammatory functions by its binding to various receptors or ligands. HMGB1 may trigger pro-inflammatory macrophages during the onset of inflammation, but monocytes may be skewed to anti-inflammatory macrophages as C1q levels rise and bind to LAIR-1. It is not clear how both pro- and anti-inflammatory responses possibly depend on its concentration. HMGB1 is abundant in the microenvironment in many chronic diseases such as Alzheimer’s disease (64), atherosclerosis (65), seizures (66, 67), and cancers (68, 69). Chronic activation of the classical complement pathway, either by deposition of HMGB1 or through its cooperative interaction with endogenous molecules such as Aβ (64), could trigger sterile inflammation and accelerate disease progression. We propose a novel Ab-independent classical complement pathway, promoted by HMGB1 binding to C1q, which augments the inflammatory process. HMGB1-mediated sterile inflammation by complement activation is, therefore, an exciting therapeutic target in chronic inflammatory conditions. Our results suggest that HMGB1 neutralization exhibits promising potential as a therapeutic strategy toward sterile inflammation caused by complement activation.

## Ethics Statement

Animal procedures were carried out according to a protocol approved by the Institutional Animal Care and Use Committee (IACUC) and the Institutional Biosafety Committee of the Yonsei Laboratory Animal Research Center (YLARC, 2010-0392) and the Feinstein Institute for Medical Research (FIMR, 2013-021), Manhasset, NY, USA. Animals were allowed to acclimate for at least 2 weeks prior to initiating the experiment. All animals were housed in standard conditions (room temperature 22°C with a 12-h light–dark cycle) with access to regular chow and water.

## Author Contributions

SY, MS, SE, and JS planned the research, performed, and analyzed data; IH, MS, MG, HS, and ES produced recombinant HMGB1 variant proteins and analyzed data; JY and JE contributed to the MCAO mouse study. JE, BD, and JS designed, interpreted and coordinated the study, and wrote the manuscript; all authors reviewed and approved the final manuscript.

## Conflict of Interest Statement

The authors declare that the research was conducted in the absence of any commercial or financial relationships that could be construed as a potential conflict of interest.

## References

[1] Andersson U Tracey KJ. HMGB1 is a therapeutic target for sterile inflammation and infection. Annu Rev Immunol (2011) 29:139–62. 10.1146/annurev-immunol-030409-101323 21219181 PMC4536551

[2] Yang H Antoine DJ Andersson U Tracey KJ. The many faces of HMGB1: molecular structure-functional activity in inflammation, apoptosis, and chemotaxis. J Leukoc Biol (2013) 93(6):865–73. 10.1189/jlb.1212662 23446148 PMC4051189

[3] Bonaldi T Talamo F Scaffidi P Ferrera D Porto A Bachi A Monocytic cells hyperacetylate chromatin protein HMGB1 to redirect it towards secretion. EMBO J (2003) 22(20):5551–60. 10.1093/emboj/cdg516 14532127 PMC213771

[4] Youn JH Shin JS. Nucleocytoplasmic shuttling of HMGB1 is regulated by phosphorylation that redirects it toward secretion. J Immunol (2006) 177(11):7889–97. 10.4049/jimmunol.177.11.7889 17114460

[5] Scaffidi P Misteli T Bianchi ME. Release of chromatin protein HMGB1 by necrotic cells triggers inflammation. Nature (2002) 418(6894):191–5. 10.1038/nature00858 12110890

[6] Wang H Bloom O Zhang M Vishnubhakat JM Ombrellino M Che J HMG-1 as a late mediator of endotoxin lethality in mice. Science (1999) 285(5425):248–51. 10.1126/science.285.5425.248 10398600

[7] Ombrellino M Wang H Ajemian MS Talhouk A Scher LA Friedman SG Increased serum concentrations of high-mobility-group protein 1 in haemorrhagic shock. Lancet (1999) 354(9188):1446–7. 10.1016/S0140-6736(99)02658-6 10543678

[8] Sunden-Cullberg J Norrby-Teglund A Rouhiainen A Rauvala H Herman G Tracey KJ Persistent elevation of high mobility group box-1 protein (HMGB1) in patients with severe sepsis and septic shock. Crit Care Med (2005) 33(3):564–73. 10.1097/01.CCM.0000155991.88802.4D 15753748

[9] Yang H Ochani M Li J Qiang X Tanovic M Harris HE Reversing established sepsis with antagonists of endogenous high-mobility group box 1. Proc Natl Acad Sci U S A (2004) 101(1):296–301. 10.1073/pnas.2434651100 14695889 PMC314179

[10] Liliensiek B Weigand MA Bierhaus A Nicklas W Kasper M Hofer S Receptor for advanced glycation end products (RAGE) regulates sepsis but not the adaptive immune response. J Clin Invest (2004) 113(11):1641–50. 10.1172/JCI200418704 15173891 PMC419481

[11] Hori O Brett J Slattery T Cao R Zhang J Chen JX The receptor for advanced glycation end products (RAGE) is a cellular binding site for amphoterin. Mediation of neurite outgrowth and co-expression of rage and amphoterin in the developing nervous system. J Biol Chem (1995) 270(43):25752–61. 10.1074/jbc.270.43.25752 7592757

[12] Park JS Svetkauskaite D He Q Kim JY Strassheim D Ishizaka A Involvement of toll-like receptors 2 and 4 in cellular activation by high mobility group box 1 protein. J Biol Chem (2004) 279(9):7370–7. 10.1074/jbc.M306793200 14660645

[13] Salmivirta M Rauvala H Elenius K Jalkanen M. Neurite growth-promoting protein (amphoterin, p30) binds syndecan. Exp Cell Res (1992) 200(2):444–51. 10.1016/0014-4827(92)90194-D 1369684

[14] Yang H Hreggvidsdottir HS Palmblad K Wang H Ochani M Li J A critical cysteine is required for HMGB1 binding to toll-like receptor 4 and activation of macrophage cytokine release. Proc Natl Acad Sci U S A (2010) 107(26):11942–7. 10.1073/pnas.1003893107 20547845 PMC2900689

[15] Yang H Wang H Ju Z Ragab AA Lundback P Long W MD-2 is required for disulfide HMGB1-dependent TLR4 signaling. J Exp Med (2015) 212(1):5–14. 10.1084/jem.20141318 25559892 PMC4291531

[16] Youn JH Oh YJ Kim ES Choi JE Shin JS High mobility group box 1 protein binding to lipopolysaccharide facilitates transfer of lipopolysaccharide to CD14 and enhances lipopolysaccharide-mediated TNF-a production in human monocytes. J Immunol (2008) 180(7):5067–74. 10.4049/jimmunol.180.7.5067 18354232

[17] Kwak MS Lim M Lee YJ Lee HS Kim YH Youn JH HMGB1 binds to lipoteichoic acid and enhances TNF-alpha and IL-6 production through HMGB1-mediated transfer of lipoteichoic acid to CD14 and TLR2. J Innate Immun (2015) 7(4):405–16. 10.1159/000369972 25660311 PMC6738877

[18] Sha Y Zmijewski J Xu Z Abraham E. HMGB1 develops enhanced proinflammatory activity by binding to cytokines. J Immunol (2008) 180(4):2531–7. 10.4049/jimmunol.180.4.2531 18250463

[19] Schiraldi M Raucci A Munoz LM Livoti E Celona B Venereau E HMGB1 promotes recruitment of inflammatory cells to damaged tissues by forming a complex with CXCL12 and signaling *via* CXCR4. J Exp Med (2012) 209(3):551–63. 10.1084/jem.20111739 22370717 PMC3302219

[20] Tian J Avalos AM Mao SY Chen B Senthil K Wu H Toll-like receptor 9-dependent activation by DNA-containing immune complexes is mediated by HMGB1 and RAGE. Nat Immunol (2007) 8(5):487–96. 10.1038/ni0707-780b 17417641

[21] Rouhiainen A Tumova S Valmu L Kalkkinen N Rauvala H Pivotal advance: analysis of proinflammatory activity of highly purified eukaryotic recombinant HMGB1 (amphoterin). J Leukoc Biol (2007) 81(1):49–58. 10.1189/jlb.0306200 16980512

[22] Walport MJ Complement. First of two parts. N Engl J Med (2001) 344(14):1058–66. 10.1056/NEJM200104053441406 11287977

[23] Walport MJ Complement. Second of two parts. N Engl J Med (2001) 344(15):1140–4. 10.1056/NEJM200104123441506 11297706

[24] Ricklin D Hajishengallis G Yang K Lambris JD. Complement: a key system for immune surveillance and homeostasis. Nat Immunol (2010) 11(9):785–97. 10.1038/ni.1923 20720586 PMC2924908

[25] Kang YH Tan LA Carroll MV Gentle ME Sim RB. Target pattern recognition by complement proteins of the classical and alternative pathways. Adv Exp Med Biol (2009) 653:117–28. 10.1007/978-1-4419-0901-5_8 19799115

[26] Paidassi H Tacnet-Delorme P Garlatti V Darnault C Ghebrehiwet B Gaboriaud C C1q binds phosphatidylserine and likely acts as a multiligand-bridging molecule in apoptotic cell recognition. J Immunol (2008) 180(4):2329–38. 10.4049/jimmunol.180.4.2329 18250442 PMC2632962

[27] Bohana-Kashtan O Ziporen L Donin N Kraus S Fishelson Z. Cell signals transduced by complement. Mol Immunol (2004) 41(6–7):583–97. 10.1016/j.molimm.2004.04.007 15219997

[28] Rogers J Cooper NR Webster S Schultz J McGeer PL Styren SD Complement activation by beta-amyloid in Alzheimer disease. Proc Natl Acad Sci U S A (1992) 89(21):10016–20. 10.1073/pnas.89.21.10016 1438191 PMC50268

[29] Ito T Kawahara K Okamoto K Yamada S Yasuda M Imaizumi H Proteolytic cleavage of high mobility group box 1 protein by thrombin-thrombomodulin complexes. Arterioscler Thromb Vasc Biol (2008) 28(10):1825–30. 10.1161/ATVBAHA.107.150631 18599803

[30] Aida Y Pabst MJ. Removal of endotoxin from protein solutions by phase separation using Triton X-114. J Immunol Methods (1990) 132(2):191–5. 10.1016/0022-1759(90)90029-U 2170533

[31] Harboe M Thorgersen EB Mollnes TE. Advances in assay of complement function and activation. Adv Drug Deliv Rev (2011) 63(12):976–87. 10.1016/j.addr.2011.05.010 21664392

[32] Kaplan MH Volanakis JE Interaction of C-reactive protein complexes with the complement system. I. Consumption of human complement associated with the reaction of C-reactive protein with pneumococcal C-polysaccharide and with the choline phosphatides, lecithin and sphingomyelin. J Immunol (1974) 112(6):2135–47. 4151108

[33] Bhakdi S Torzewski M Paprotka K Schmitt S Barsoom H Suriyaphol P Possible protective role for C-reactive protein in atherogenesis: complement activation by modified lipoproteins halts before detrimental terminal sequence. Circulation (2004) 109(15):1870–6. 10.1161/01.CIR.0000124228.08972.26 15037531

[34] Abbott NJ Ronnback L Hansson E. Astrocyte-endothelial interactions at the blood–brain barrier. Nat Rev Neurosci (2006) 7(1):41–53. 10.1038/nrn1824 16371949

[35] Kim JH Yenari MA Giffard RG Cho SW Park KA Lee JE. Agmatine reduces infarct area in a mouse model of transient focal cerebral ischemia and protects cultured neurons from ischemia-like injury. Exp Neurol (2004) 189(1):122–30. 10.1016/j.expneurol.2004.05.029 15296842

[36] Singhal R Ganey PE Roth RA. Complement activation in acetaminophen-induced liver injury in mice. J Pharmacol Exp Ther (2012) 341(2):377–85. 10.1124/jpet.111.189837 22319198 PMC3336815

[37] Kang YS Do Y Lee HK Park SH Cheong C Lynch RM A dominant complement fixation pathway for pneumococcal polysaccharides initiated by SIGN-R1 interacting with C1q. Cell (2006) 125(1):47–58. 10.1016/j.cell.2006.01.046 16615889

[38] Doekes G van Es LA Daha MR. Activation of C1 by soluble IgG aggregates as detected by a novel one-step hemolytic assay that specifically measures the proenzyme form of C1s. J Immunol (1983) 131(4):1924–9. 6604753

[39] Wang Q Rozelle AL Lepus CM Scanzello CR Song JJ Larsen DM Identification of a central role for complement in osteoarthritis. Nat Med (2011) 17(12):1674–9. 10.1038/nm.2543 22057346 PMC3257059

[40] Bianchi ME. HMGB1 loves company. J Leukoc Biol (2009) 86(3):573–6. 10.1189/jlb.1008585 19414536

[41] Charchaflieh J Wei J Labaze G Hou YJ Babarsh B Stutz H The role of complement system in septic shock. Clin Dev Immunol (2012) 2012:407324. 10.1155/2012/407324 23049598 PMC3459296

[42] Dillon SP D’Souza A Kurien BT Scofield RH. Systemic lupus erythematosus and C1q: a quantitative ELISA for determining C1q levels in serum. Biotechnol J (2009) 4(8):1210–4. 10.1002/biot.200800273 19370710 PMC2829988

[43] Moskovich O Herzog LO Ehrlich M Fishelson Z. Caveolin-1 and dynamin-2 are essential for removal of the complement C5b-9 complex *via* endocytosis. J Biol Chem (2012) 287(24):19904–15. 10.1074/jbc.M111.333039 22528500 PMC3370175

[44] Li Y Lin F. Mesenchymal stem cells are injured by complement after their contact with serum. Blood (2012) 120(17):3436–43. 10.1182/blood-2012-03-420612 22966167 PMC3482856

[45] Zhang J Takahashi HK Liu K Wake H Liu R Maruo T Anti-high mobility group box-1 monoclonal antibody protects the blood–brain barrier from ischemia-induced disruption in rats. Stroke (2011) 42(5):1420–8. 10.1161/STROKEAHA.110.598334 21474801

[46] Kishore U Gaboriaud C Waters P Shrive AK Greenhough TJ Reid KB C1q and tumor necrosis factor superfamily: modularity and versatility. Trends Immunol (2004) 25(10):551–61. 10.1016/j.it.2004.08.006 15364058

[47] Venereau E Casalgrandi M Schiraldi M Antoine DJ Cattaneo A De Marchis F Mutually exclusive redox forms of HMGB1 promote cell recruitment or proinflammatory cytokine release. J Exp Med (2012) 209(9):1519–28. 10.1084/jem.20120189 22869893 PMC3428943

[48] Son M Porat A He M Suurmond J Santiago-Schwarz F Andersson U C1q and HMGB1 reciprocally regulate human macrophage polarization. Blood (2016) 128(18):2218–28. 10.1182/blood-2016-05-719757 27683415 PMC5095756

[49] Nelson SD Molecular mechanisms of the hepatotoxicity caused by acetaminophen. Semin Liver Dis (1990) 10(4):267–78. 10.1055/s-2008-1040482 2281334

[50] Jaeschke H McGill MR Williams CD Ramachandran A Current issues with acetaminophen hepatotoxicity—a clinically relevant model to test the efficacy of natural products. Life Sci (2011) 88(17–18):737–45. 10.1016/j.lfs.2011.01.025 21296090 PMC3076526

[51] Martin-Murphy BV Holt MP Ju C. The role of damage associated molecular pattern molecules in acetaminophen-induced liver injury in mice. Toxicol Lett (2010) 192(3):387–94. 10.1016/j.toxlet.2009.11.016 19931603 PMC2822049

[52] Chen GY Tang J Zheng P Liu Y. CD24 and Siglec-10 selectively repress tissue damage-induced immune responses. Science (2009) 323(5922):1722–5. 10.1126/science.1168988 19264983 PMC2765686

[53] Antoine DJ Dear JW Lewis PS Platt V Coyle J Masson M Mechanistic biomarkers provide early and sensitive detection of acetaminophen-induced acute liver injury at first presentation to hospital. Hepatology (2013) 58(2):777–87. 10.1002/hep.26294 23390034 PMC3842113

[54] Kim JB Lim CM Yu YM Lee JK. Induction and subcellular localization of high-mobility group box-1 (HMGB1) in the postischemic rat brain. J Neurosci Res (2008) 86(5):1125–31. 10.1002/jnr.21555 17975839

[55] Rittirsch D Flierl MA Nadeau BA Day DE Huber-Lang M Mackay CR Functional roles for C5a receptors in sepsis. Nat Med (2008) 14(5):551–7. 10.1038/nm1753 18454156 PMC2753858

[56] Mahajan SD Parikh NU Woodruff TM Jarvis JN Lopez M Hennon T C5a alters blood–brain barrier integrity in a human *in vitro* model of systemic lupus erythematosus. Immunology (2015) 146(1):130–43. 10.1111/imm.12489 26059553 PMC4552508

[57] Jacob A Hack B Chiang E Garcia JG Quigg RJ Alexander JJ. C5a alters blood–brain barrier integrity in experimental lupus. FASEB J (2010) 24(6):1682–8. 10.1096/fj.09-138834 20065106 PMC2874478

[58] Wang C Wang H Hao J Chang DY Zhao MH Chen M. Involvement of high mobility group box 1 in the activation of C5a-primed neutrophils induced by ANCA. Clin Immunol (2015) 159(1):47–57. 10.1016/j.clim.2015.04.008 25934387

[59] Flierl MA Stahel PF Rittirsch D Huber-Lang M Niederbichler AD Hoesel LM Inhibition of complement C5a prevents breakdown of the blood–brain barrier and pituitary dysfunction in experimental sepsis. Crit Care (2009) 13(1):R12. 10.1186/cc7710 19196477 PMC2688129

[60] Webster S Glabe C Rogers J. Multivalent binding of complement protein C1Q to the amyloid beta-peptide (A beta) promotes the nucleation phase of A beta aggregation. Biochem Biophys Res Commun (1995) 217(3):869–75. 10.1006/bbrc.1995.2852 8554610

[61] Li J Kokkola R Tabibzadeh S Yang R Ochani M Qiang X Structural basis for the proinflammatory cytokine activity of high mobility group box 1. Mol Med (2003) 9(1–2):37–45. 12765338 PMC1430376

[62] Tsung A Sahai R Tanaka H Nakao A Fink MP Lotze MT The nuclear factor HMGB1 mediates hepatic injury after murine liver ischemia–reperfusion. J Exp Med (2005) 201(7):1135–43. 10.1084/jem.20042614 15795240 PMC2213120

[63] Lindvall B Bengtsson A Ernerudh J Eriksson P Subclinical myositis is common in primary Sjogren’s syndrome and is not related to muscle pain. J Rheumatol (2002) 29(4):717–25. 11950012

[64] Kallijarvi J Haltia M Baumann MH. Amphoterin includes a sequence motif which is homologous to the Alzheimer’s beta-amyloid peptide (Abeta), forms amyloid fibrils *in vitro*, and binds avidly to Abeta. Biochemistry (2001) 40(34):10032–7. 10.1021/bi002095n 11513581

[65] Kalinina N Agrotis A Antropova Y DiVitto G Kanellakis P Kostolias G Increased expression of the DNA-binding cytokine HMGB1 in human atherosclerotic lesions: role of activated macrophages and cytokines. Arterioscler Thromb Vasc Biol (2004) 24(12):2320–5. 10.1161/01.ATV.0000145573.36113.8a 15374849

[66] Maroso M Balosso S Ravizza T Liu J Aronica E Iyer AM Toll-like receptor 4 and high-mobility group box-1 are involved in ictogenesis and can be targeted to reduce seizures. Nat Med (2010) 16(4):413–9. 10.1038/nm.2127 20348922

[67] Choi J Min HJ Shin JS. Increased levels of HMGB1 and pro-inflammatory cytokines in children with febrile seizures. J Neuroinflammation (2011) 8:135. 10.1186/1742-2094-8-135 21989210 PMC3210097

[68] Apetoh L Ghiringhelli F Tesniere A Obeid M Ortiz C Criollo A Toll-like receptor 4-dependent contribution of the immune system to anticancer chemotherapy and radiotherapy. Nat Med (2007) 13(9):1050–9. 10.1038/nm1622 17704786

[69] Sims GP Rowe DC Rietdijk ST Herbst R Coyle AJ. HMGB1 and RAGE in inflammation and cancer. Annu Rev Immunol (2010) 28:367–88. 10.1146/annurev.immunol.021908.132603 20192808

