# Supplementary material for: High-Mobility Group Box 1-Induced Complement Activation Causes Sterile Inflammation
Source: Front Immunol. 2018 Apr 11;9:705. doi: 10.3389/fimmu.2018.00705 (PMC5904255; doi:10.3389/fimmu.2018.00705)
Supplement: Supplementary file 1 [file Data_Sheet_1.PDF]

High Mobility Group Box 1-Induced Complement Activation Causes Sterile Inflammation

Sook Young Kim,[1,7] Myoungsun Son,[2,7] Sang Eun Lee,[1,7] In Ho Park,[1,3] Man Sup Kwak,[1] Myeonggil Han,[1] Hyun Sook Lee,[1] Eun Sook Kim,[1] Jae-Young Kim,[4] Jong Eun Lee,[4] Ji Eun Choi,[5] Betty Diamond,[2] and Jeon-Soo Shin[1,3,6,*]

[1]Department of Microbiology, Yonsei University College of Medicine, Seoul, South Korea, [2]The Center for Autoimmune Musculoskeletal and Hematopoietic Diseases, The Feinstein Institute for Medical Research, 350 Community Drive, Manhasset, NY, United States, [3]Severance Biomedical Science Institute and Institute for Immunology and Immunological Diseases, Yonsei University College of Medicine, Seoul, South Korea, [4]Department of Anatomy, Yonsei University College of Medicine, Seoul, South Korea, [5]Department of Pediatrics, Seoul National University Boramae Hospital, Seoul National University College of Medicine, Seoul, South Korea, [6]Center for Nanomedicine, Institute for Basic Science (IBS), Seoul, South Korea;

[7] These authors contributed equally to this work.

*Correspondence: Jeon-Soo Shin, E-mail: jsshin6203@yuhs.ac

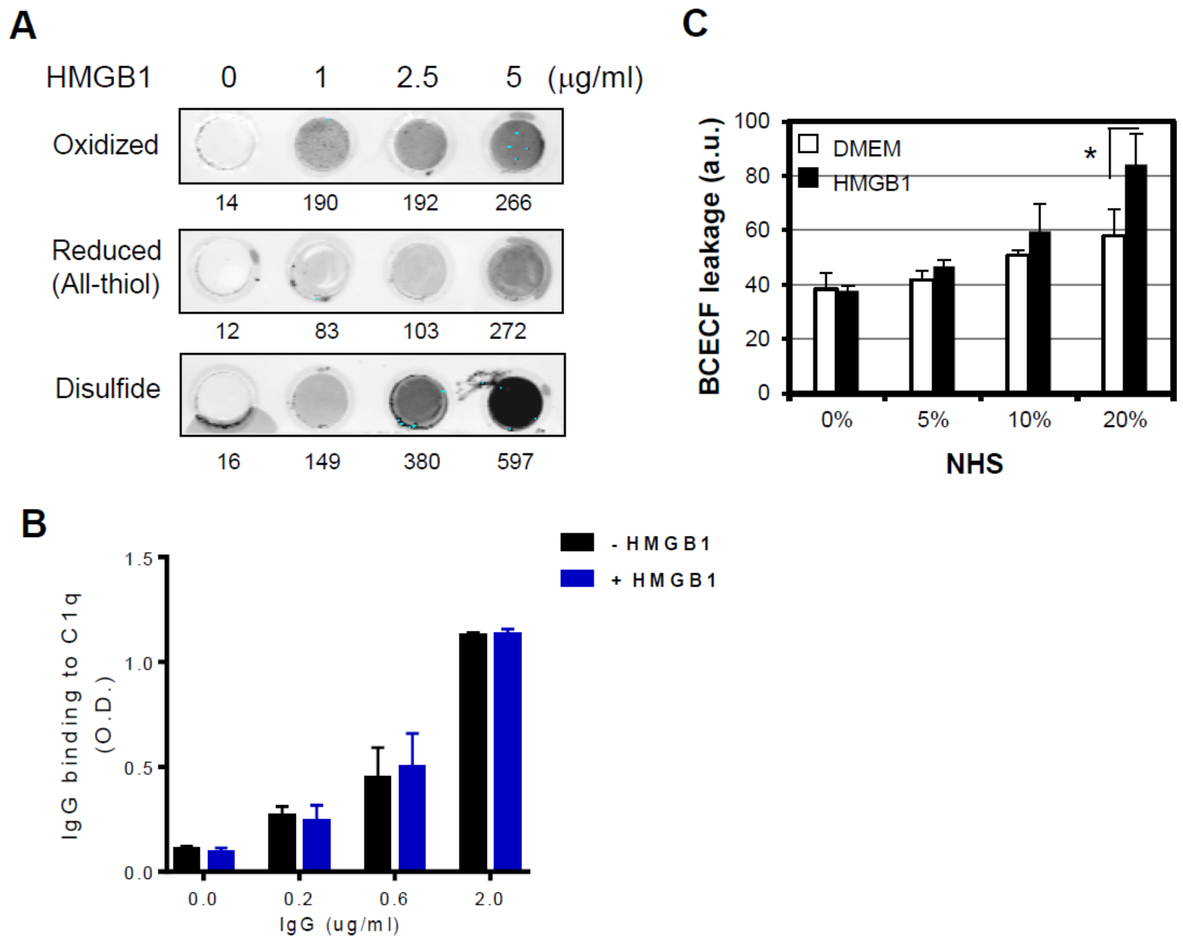

Supplementary Figure S1.
(A) Binding of C1q to HMGB1 in different redox state. HMGB1 in different concentration was loaded on nitrocellulose membrane in slot blot apparatus, and blocked in 2% skim milk for 1 h. Biotin-C1q at 10 µg/ml concentration was added for 2 h at RT followed by washing three times with 0.05% PBST. IR800-STA (1:10000) in PBST was added. The dot intensities were measured and PBS was used for negative control.

(B) HMGB1 does not alter the interaction between IgG and C1q. C1q (25 µg/ml) was coated in costar half volume plate in PBS overnight at 4°C and blocked in 1 % BSA in PBS for 1hr at RT. IgG antibodies in 1 % BSA in PBS (2 µg/ml or 0.2 µg/ml) with or without HMGB1 (50 µg/ml) were incubated for 1 h at RT. Plate bound IgG were detected by AP-conjugated anti-mouse IgG (Southern biotech) and phosphate substrate. Data are representative of two independent experiments.

(C) Membrane integrity assay. bEnd.3 cells (2.2×10^5 cells/well) were pre-incubated with 3 µM of BCECF (Invitrogen) for 30 min at 37°C and incubated with different amounts of NHS in the presence or absence of 5 µg/ml reduced form of HMGB1 for 2 h. Cells were washed and incubated with different amounts of NHS in the presence or absence of 5 µg/ml HMGB1

for 2 h. BCECF release into supernatant was measured using a fluorescence microtiter plate reader (Molecular Devices) with excitation and emission wavelengths of 485 nm and 538 nm. N=3, * *P* <0.05 by paired t-test.
